# Supplementary material for: A unique Toxoplasma gondii haplotype accompanied the global expansion of cats
Source: Nat Commun. 2022 Oct 1;13:5778. doi: 10.1038/s41467-022-33556-7 (PMC9526699; doi:10.1038/s41467-022-33556-7)
Supplement: Supplementary file 1 — Supplementary Information [file 41467_2022_33556_MOESM1_ESM.pdf]

SUPPLEMENTARY INFORMATION

A unique *Toxoplasma gondii* haplotype accompanied the global expansion of cats.

Supplementary Note 1: Identifying clonal lineages 2
Supplementary Note 2: Parasitic culture 2
Supplementary Note 3: Mutation rate and generation time estimation 3
Supplementary Fig. 1 7
Supplementary Fig. 2 21
Supplementary Fig. 3 22
Supplementary Fig. 4 23
Supplementary Fig. 5. 25
Supplementary Fig. 6. 39
Supplementary Fig. 7. 40
Supplementary Fig. 8. 41
Supplementary Fig. 9. 43
Supplementary Fig. 10 44
Supplementary Fig. 11 47
Supplementary Table 1 49
Supplementary Table 2 50
Supplementary Discussion 1 50
Supplementary Discussion 2 50
Supplementary Discussion 3 51
Supplementary Discussion 4 53
Supplementary Discussion 5 54
REFERENCES 54

Supplementary Note 1: Identifying clonal lineages

Strict clonal multiplication in a population is characterized by gradual accumulation of random mutations over generations[1]. At the opposite, recombination with a distinct population introduces highly divergent genomic regions, leading to a sharp rise in genetic distances between individuals. The mlg.filter function of poppr R package exploits this property by enabling to define a threshold that delimits genetic distances resulting from the gradual accumulation of random mutations (small genetic distances) within a population from genetic distances resulting from recombination with a divergent population (large genetic distances). This threshold is expected to correspond to the maximal genetic distance resulting only from mutation accumulation (within a clonal lineage), and above which genetic distances are rather explained by recombination. The function mlg.filter gradually collapses genomes based on genetic distance (along the horizontal axis) using three different clustering algorithms in order to define clonal lineages boundaries. A sharp drop in the number of uncollapsed genomes is observed when collapsing genomes separated by a genetic distance < 0.01 (Supplementary Fig. 10). At this threshold value, all strains separated by small genetic distances are collapsed together; we consider that they belong to the same clonal lineage. This threshold was used to generate a minimum spanning network (MSN), in which strains of the same putative clonal lineage were collapsed in single circles. Overall, few mismatches between MS-defined and genome-defined lineages were noticed, as only two genomes did not cluster with the other strains of their respective MS-defined lineages: MARTINIQUE01 (type II) and GABON08 (Africa 1). GABON02 clustered with type III strains although it had a variant allele at one of the MS typing markers (TUB2). To verify that poppr-defined lineages are true clonal lineages, we generated plots of SNPs density for each of the main lineages (type I, type II, type III, Africa 1 and Africa 4) by dividing the genome into 10kb windows (Supplementary Fig. 11). We expected to observe an even genome-wide density of SNPs in the case of strict clonality, and to observe sharp variations in the density of SNPs (designated as recombination break points) if one or more strains have inherited divergent genomic sequences following recombination with another lineage or population[2]. We found repeated sharp variations in SNP densities in plots produced for Africa 4 and type III lineages, a pattern usually observed when recombination break points are disrupting chromosomal ancestry along one or more genomes. We calculated pairwise SNP distances between the genomes of each of these two lineages. Among Africa 4 genomes (n=3), SENEGAL04 showed 9,820 SNP differences with SENEGAL14 and SENEGAL23, the latter two showing no SNP differences, indicating the presence of regions with distinct ancestry between these two groups. Type III genomes (n=20) showed a maximum of 350 SNPs differences, with the exception of USA04 (M7741) that showed 10,077 to 10,459 SNP differences with other type III genomes. By excluding this divergent genome and generating a new SNP density plot, the sharp variations in SNP densities previously observed did not recur, indicating that the excluded genome had divergent ancestry in certain chromosomal portions (Supplementary Fig. 11). The other lineages (type I, type II and Africa 1) exhibited an even distribution of SNPs across their genomes, albeit type II had a much higher SNPs density compared to the other lineages (Supplementary Table 2).

Supplementary Note 2: Parasitic culture

Each cryopreserved strain from the BRC *Toxoplasma* was intraperitoneally inoculated into two out-bred female Swiss Webster (SW) mice (1 mL/mice). Animal experimentation was approved and

accepted by the Ethics Committee for Animal Experimentation n°033 validated by the French Ministry of National Education, Higher Education and Research (Registration numbers: APAFIS#14582-2018041010294175 v2). Experimental procedures were conducted according to European guidelines for animal care (‘‘Journal Officiel des Communautés Européennes”, L358, December 18, 1986). Mice were housed in the animal facility of Limoges University under controlled conditions (20-24°C, 45-65% humidity, 12 hours light/12 hours dark cycle) with free access to standard mouse chow and tap water. All inoculated mice were monitored daily for clinical signs of toxoplasmosis during four weeks. Ill mice developing ascites were aseptically punctured for peritoneal exudates to collect live tachyzoites before being euthanized. Peritoneal exudates were washed with sterile saline solution (0.9% NaCl) at 1500 rpm for 10 minutes. After four weeks, surviving mice were tested for *T. gondii* antibodies by modified agglutination test (cut-off at 1:20 serum dilution). Seropositive mice were euthanized and brain samples were aseptically collected, rinsed in saline solution, placed in 1 ml of saline solution, and extruded through a 21-gauge needle several times, and then through a 23-gauge needle. Half of this suspension was treated by 1 ml of trypsin-EDTA solution (pre-heated at 37˚C), thoroughly shaken, and incubated at 37˚C for 3 minutes to disrupt tissue-cysts and liberate bradyzoites. The obtained suspension was then re-extruded through a 25-gauge needle several times, washed in 5 ml of Iscove’s Modified Dulbecco’s Medium (IMDM), and re-suspended in 1ml of IMDM. Each aseptically prepared mouse sample containing either tachyzoites or bradyzoites was inoculated in a Vero cell monolayer in a T175-flask. The culture medium was composed of IMDM treated with 1% of antibiotic saline solution (1000 U/ml penicillin and 100 µg streptomycin/ml in saline solution) and enriched with 2% of foetal bovine serum (FBS). Parasite growth was observed between one and five weeks post-initial inoculation but lasted between two weeks and 5 months. When parasitic growth was sufficient (> 40 tachyzoites at 40x magnification), the T-175 flask was vigorously shaken, the culture media collected and spun at 380 rpm for 5 min to pellet the large debris. The supernatant was then pelleted with a faster spin of 1500 rpm for 10 min. Then the supernatant was discarded, the tachyzoites pellet re-suspended with 2 ml of PBS free of Ca2+ and Mg2+, and the mix was homogenized through several passages in a 25-gauge needle, before adding 10 ml of PBS and homogenizing the mix with gentle shaking. The tachyzoite suspension was then filtrated through 3.0 micron polycarbonate filters, spun at 1500 rpm for 10 min, before reducing PBS volume to 1 ml and re-suspending the pellet in this volume. Lastly, the tachyzoite suspension was spun at 4500 rpm for 5 min and the volume reduced to 200 µl.

Supplementary Note 3: Mutation rate and generation time estimation

Estimation of single nucleotide substitution per mitosis

RH strain

The laboratory of Parasitology of the Limoges University Hospital Centre carried out *in vivo* culture of the RH strain (type I lineage) to produce *T. gondii* antigen for purposes of human diagnosis. This strain was maintained during 30 years (from January 1989 to march 11th 2019) through successive passages in outbred mice. This strain is virulent for the mouse as its growth cannot be controlled by its immune system, leading to the development of an ascites rich in tachyzoites in 48h post-inoculation. Three passages in mice were carried out each week; the ascites was punctured from

infected mice and after being euthanized, the number of tachyzoites was counted, and 1,000,000 to 1,500,000 tachyzoites were again inoculated to two new mice.

In total, the RH strain was cultured *in vivo* during ~259,482 hours (~30 years). Assuming a division time of 5 hours [3], we calculated that ~ 51,896 mitotic divisions occurred during this period. In January 1989, tachyzoites were collected from an infected mouse, filtrated and stored at -80°C. The same protocol was followed for tachyzoites collected from an infected mouse in March 2019, with the addition of a step of cell culture to obtain more parasitic DNA. For these two samples, we applied the same protocol used for sequencing, variant calling and annotation of field isolates (with the exception of using per default mapping configuration in BWE). We determined the number of mutations that have accumulated over this period by comparing the two strains. Eighty-one SNPs were found across the 13 nuclear chromosomes (63,973,855 bases) following all filtration steps, and 67 SNPs were validated after manual curation on IGV 2.9.4.

Estimation of the mutation rate of RH strain based on whole nuclear genome data:

Mutation rate per mitosis: $\frac{67}{51{,}896} = 1.3\text{x}10^{-3}$

Mutation rate per mitosis per site: $\frac{67}{51{,}896 * 63{,}973{,}855} = 2.0\text{x}10^{-11}$

There were 25 exonic (18 missense, 2 nonsense and 5 silent), 20 intronic and 22 intergenic SNPs. This higher proportion of non-synonymous *vs* synonymous mutations (Missense / Silent ratio of 3.6) is consistent with selective constraints acting on RH strain, probably due to the unnatural conditions of maintenance of this strain for many generations. In order to mitigate bias in mutation rate associated to selection, we choses to calculate the mutation rate of RH strain from the SNPs found in the intergenic regions of the genome (23,896,470 nucleotides).

Estimation of the mutation rate of RH strain based on intergenic sequence data:

Mutation rate per mitosis: $\frac{22}{51{,}896} = 4.2\text{x}10^{-4}$

Mutation rate per mitosis per site: $\frac{22}{51{,}896 * 23{,}896{,}470} = 1.8\text{x}10^{-11}$

PRU strain

For comparison purposes, we also calculated the mutation rate of PRU strain (type II lineage). This strain was also maintained during 30 years, with only 75 passages in outbred mice (2.5 passages per year in average). This strain is not virulent to mice; after an acute phase characterized by rapid parasite multiplication, the parasites form dormant intracellular tissue cysts to evade the host's immune system[4]. The acute phase of infection lasts ~21 days and can be divided into two phases[5]. First, tachyzoites carry out rapid multiplication during ten days. Tachyzoites of type II strains have a division time of 9 hours —an estimation made on ME49 strain[3]— and hence this stage involves ~27 mitoses. Then, tachyzoites differentiate into bradyzoites, which continue to multiply for 11 days, albeit at a slower rate. Bradyzoite division time has not been estimated for type II strains and we based our calculations on the estimate obtained from VEG strain (type III), which is of 15 hours (Jerome et al., 1998). Hence, this second stage of multiplication involves ~18 mitoses. Assuming a total number of 45 mitoses per passage, we calculated that 3,375 mitoses can occur during the 75

passages. We followed the same protocol as that used for RH to PRU for culture, sequencing and variant calling. We first identified 8 SNPs differentiating PRU-1989 from PRU-2019, a number than drops to 4 SNPs after manual curation in IGV.

Estimation of the mutation rate of PRU strain based on whole nuclear genome data:

Mutation rate per mitosis: $\frac{4}{3{,}375} = 1.2\text{x}10^{-3}$

Mutation rate per mitosis per site: $\frac{4}{3{,}375 * 63{,}973{,}855} = 1.8\text{x}10^{-11}$

The mutation rates of RH and PRU showed a high degree of agreement, which reinforces the robustness of our estimates. The RH strain was subjected to a much higher number of passages than the PRU strain (51,896 versus 3,375), and we therefore chose to base all subsequent calculations on the RH estimate for better precision.

Estimation of single nucleotide substitution per year

Toxoplasma gondii has a life cycle composed of three stages: in (1) the final host (cats), (2) the environment and (3) the intermediate host (mainly rodents). Several full life cycles can occur within one year.

The total number of DNA replications per year was calculated based on different assumptions about number of mitoses per stage and time to complete different stages of the life cycle.

When infected with one to few (~10) bradyzoites, cats excrete in total ~ 50 to 100 million oocysts[6]. Reaching this number of oocysts involves ~26 mitoses. Infected cats can excrete oocysts between the 3^{d} and the 21th days following infection[7–9], although most oocysts are shed between the 6th and the 13th days [6].

In the environment, the newly secreted oocyst contains a single diploid sporoblast, that undergoes a meiotic division (2N–N), followed by a mitotic division, giving rise to eight haploid nuclei. Two DNA replications occur during this stage [10]. Oocysts sporulate in the environment in 48-72 hours after their excretion; their survival depends mainly on climatic conditions, and is favoured by humidity. Although sporulated *T. gondii* oocysts can survive in the environment for 1.5 years, the median survival time varies between 27 days under dry conditions and 84 days under damp (ideal) conditions [11].

Sporulated oocysts ingested by an intermediate host differentiate into rapidly replicating tachyzoites during the first 10 days of infection [5]. Tachyzoites division time varies according to the parasitic strains[3,5]: 5 hours for RH (type I), 9 hours for ME49 (type II) and 6 hours for VEG (type III). Hence, this first stage of multiplication in the intermediate host involves a minimum of 27 mitoses (assuming a division time of 9 hours) and a maximum of 48 mitoses (assuming a division time of 5 hours). The tachyzoites then differentiate into slow replicating bradyzoites which continue to actively replicate until the end of the third week[4]. Bradyzoite division time is 15 hours[5], an estimate obtained from VEG strain (type III). Hence, this second stage of multiplication in the intermediate host involves ~18 mitoses.

The length of this stage depends on the period between the infection time and time of death. Rodent lifespans vary by species and can reach several years for some species: voles and house mice, which are common cat prey, have a median lifespan of 60-90 days[12] and 90-120 days[13], respectively. Infection can occur at any time from weaning age (~21 days[14,15]) until death. Thus, we assume on average that *T. gondii* is likely to infect rodent for:

Lower assumption: $\frac{60-21}{2} \approx 20$ days   Higher assumption: $\frac{120-21}{2} \approx 50$ days

Summary table of the duration in days of the life cycle stages

stages	minimum	lower assumption	higher assumption	maximum
final host	3	6	13	21
environment	2	27	87	547
intermediate host	1	20	50	Hundreds
**full life cycle**	**6**	**53**	**150**	**Hundreds**

By summing the estimates for the three stages, we get an estimate of 73 to 94 mitoses per full life cycle and of 2.4 to 6.9 life cycles per year. We used these estimates to calculate the mutation rate of *T. gondii* per life cycle and per year:

Mutation rate per full life cycle:

($4.2x10^{-4}$ x 73) to ($4.2x10^{-4}$ x 94) => 0.03 to 0.04

Mutation rate per full life cycle per site:

($1.8x10^{-11}$ x 73) to ($1.8x10^{-11}$ x 94) => $1.3x10^{-9}$ to $1.7x10^{-9}$

Mutation rate per year:

(0.03x 2.4) to (0.04x 6.9) => 0.07 to 0.28

Mutation rate per year per site:

($1.3x10^{-9}$ x 2.4) to ($1.7x10^{-9}$x 6.9) => $3.1x10^{-9}$ to $11.7x10^{-9}$

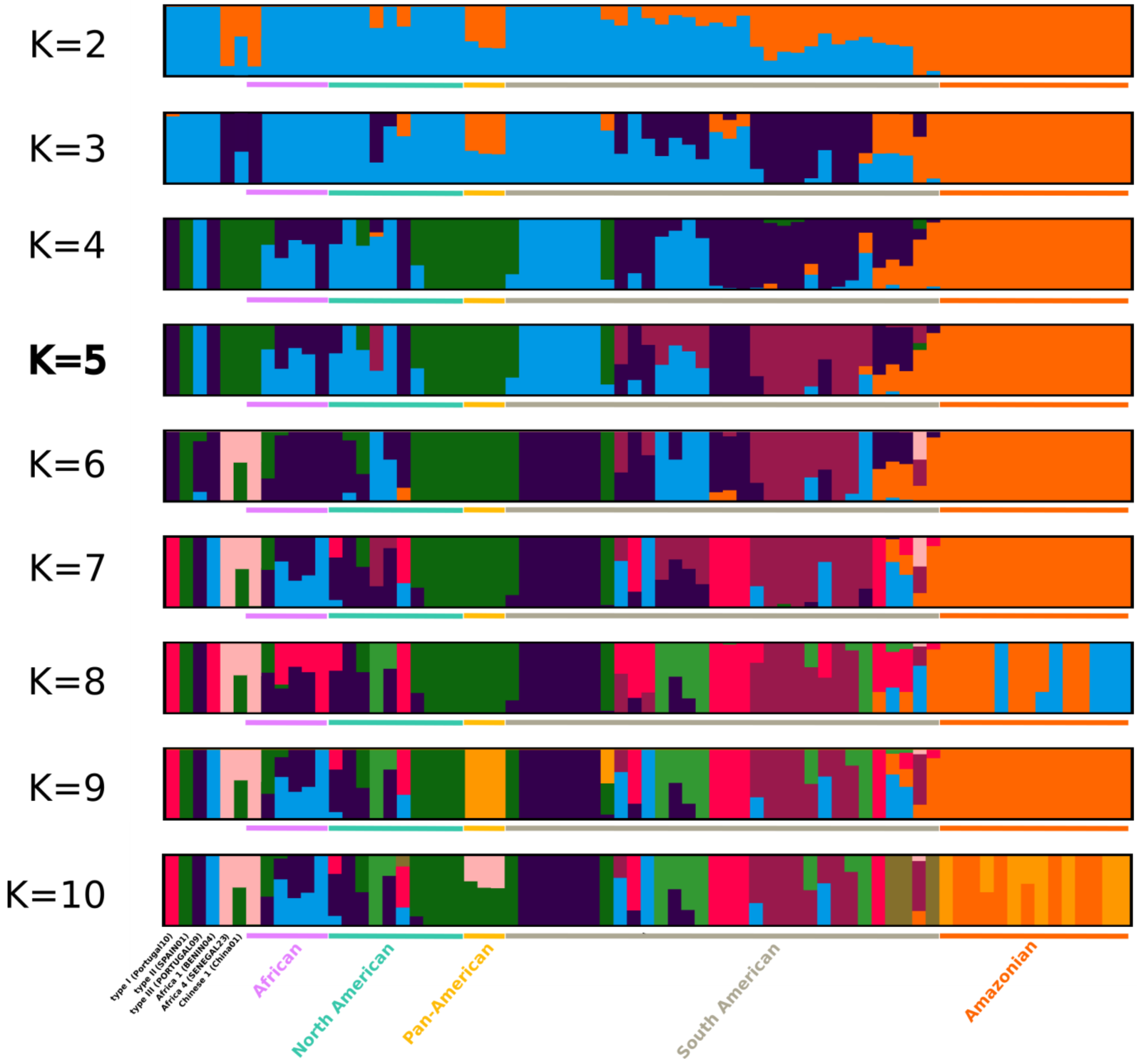

Supplementary Fig. 1. Ancestry plots showing proportions of ancestral populations for each *Toxoplasma gondii* genome for K = 2 to 10. Ancestry plots are graphically displayed using CLUMPAK[16]. Apart from samples representing the major clonal lineages (in the left), samples are ordered according to their origin.

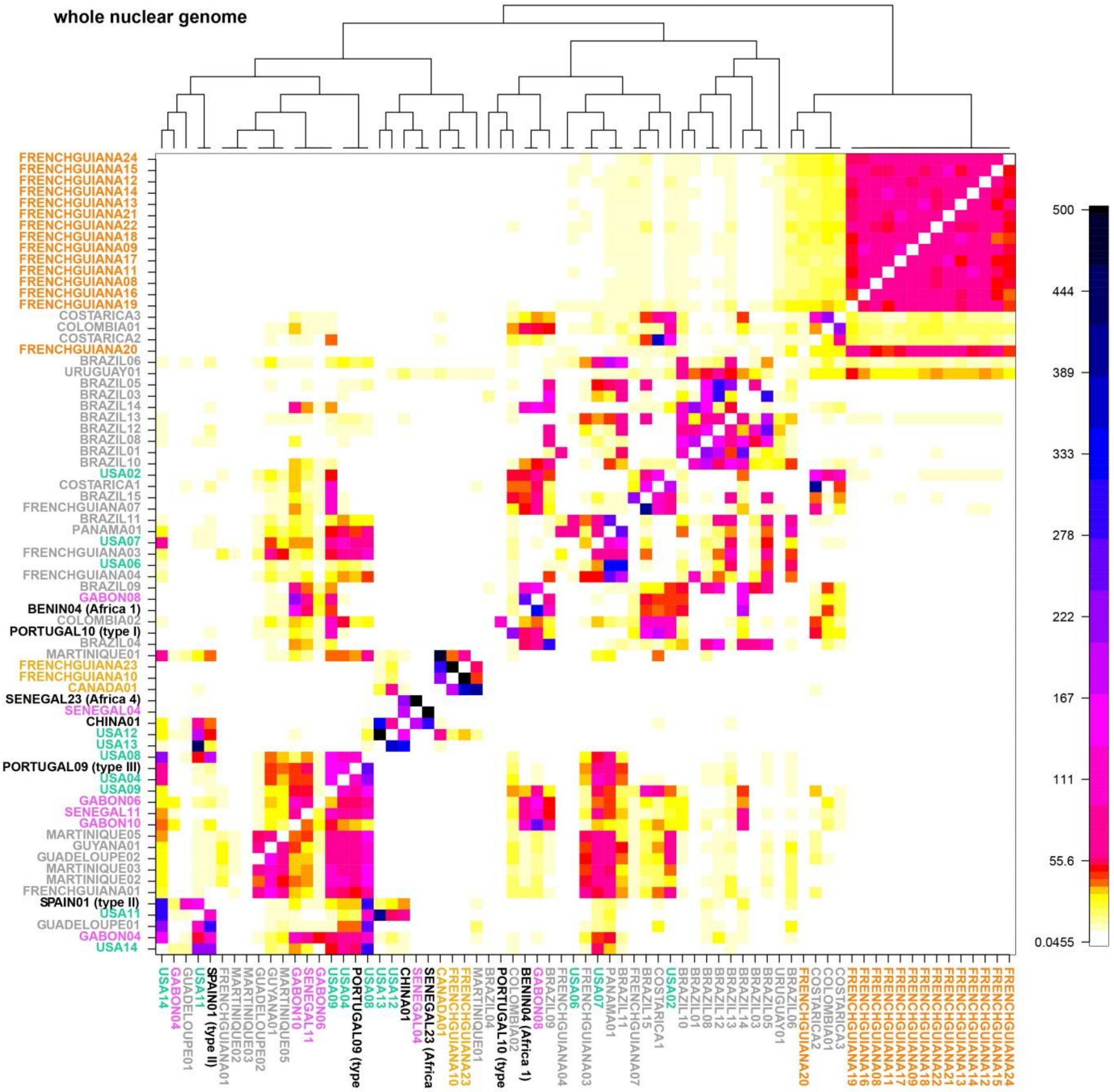

Supplementary Fig. 2a.

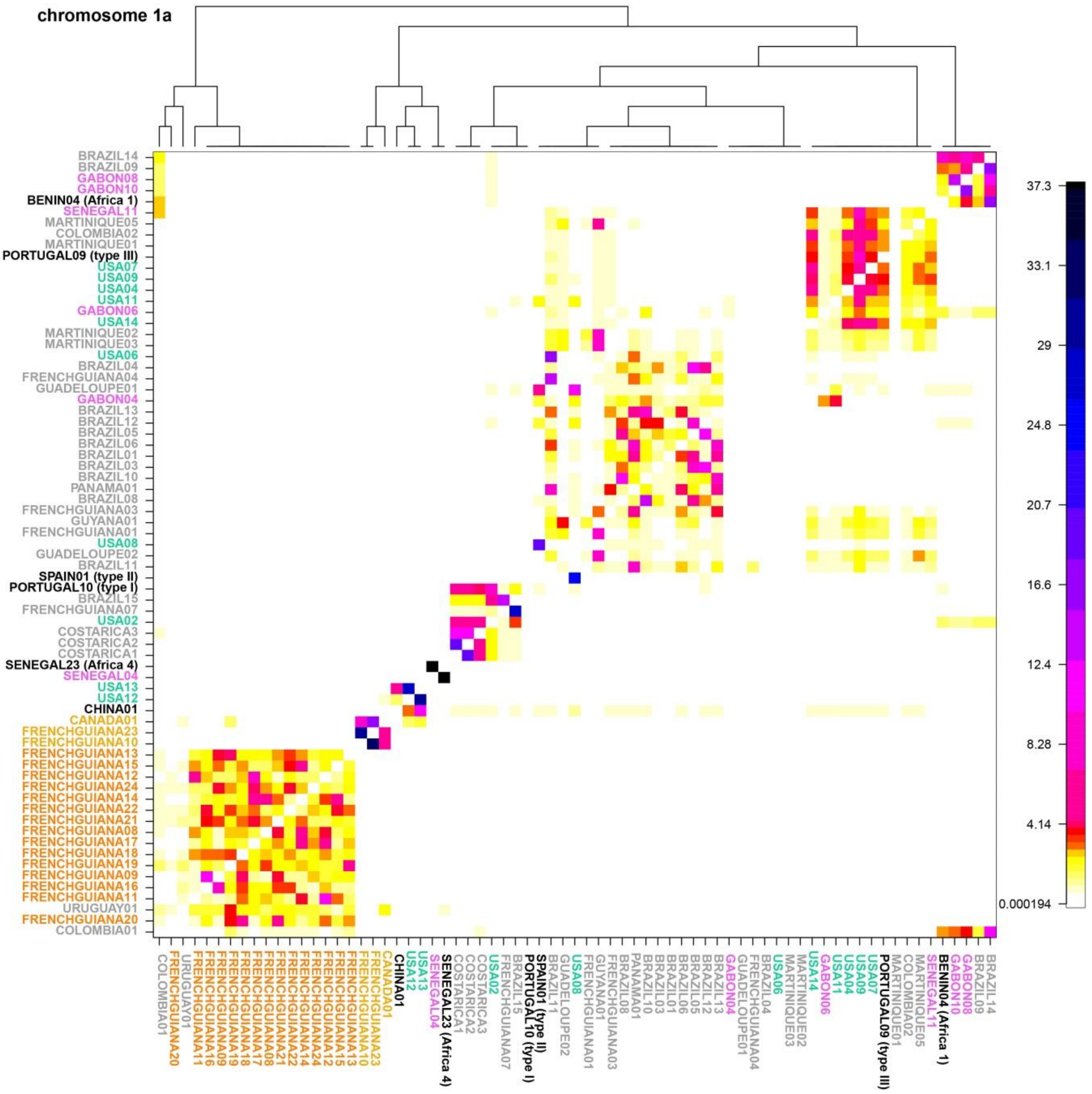

Supplementary Fig. 2b.

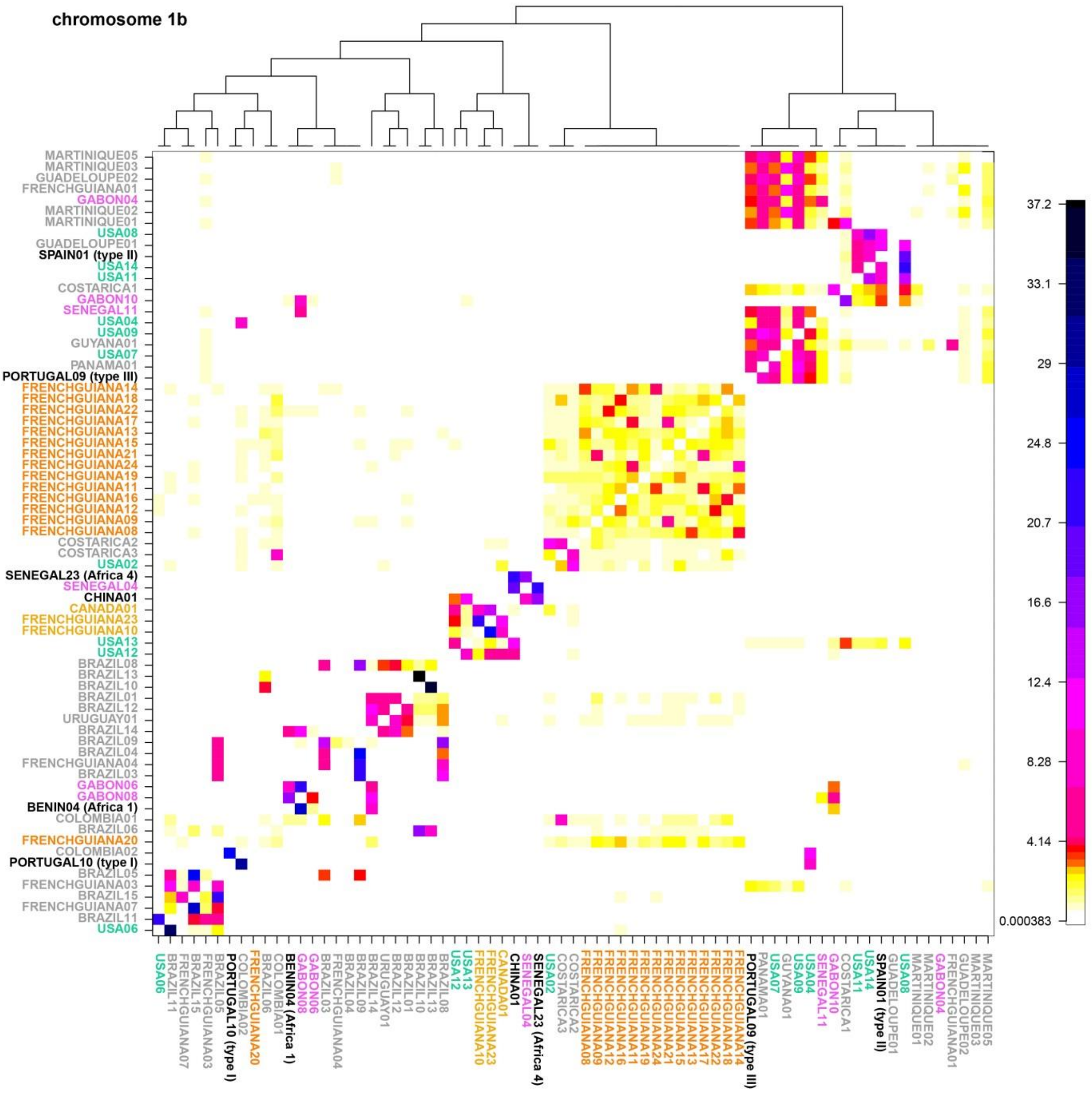

Supplementary Fig. 2c.

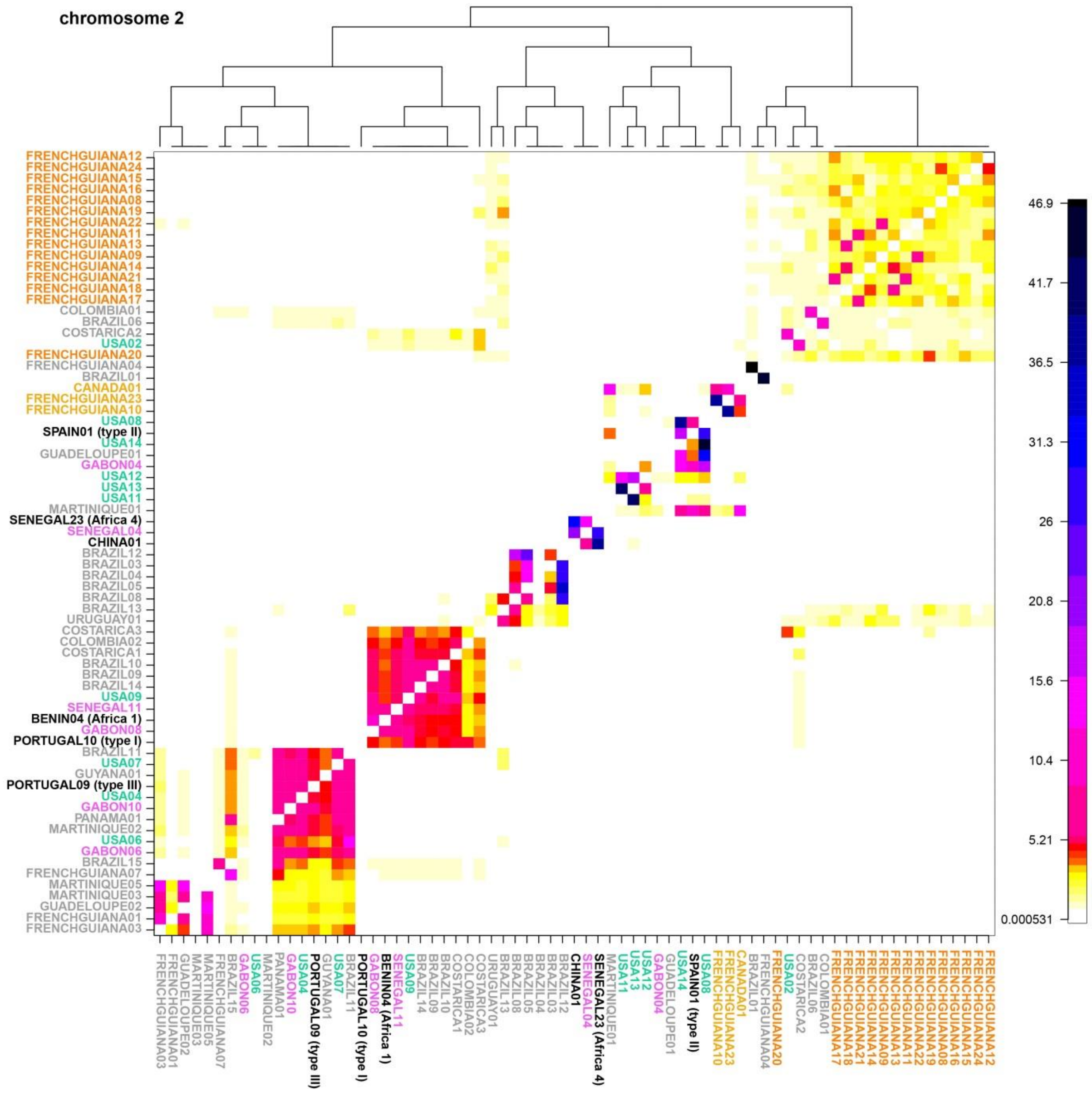

Supplementary Fig. 2d.

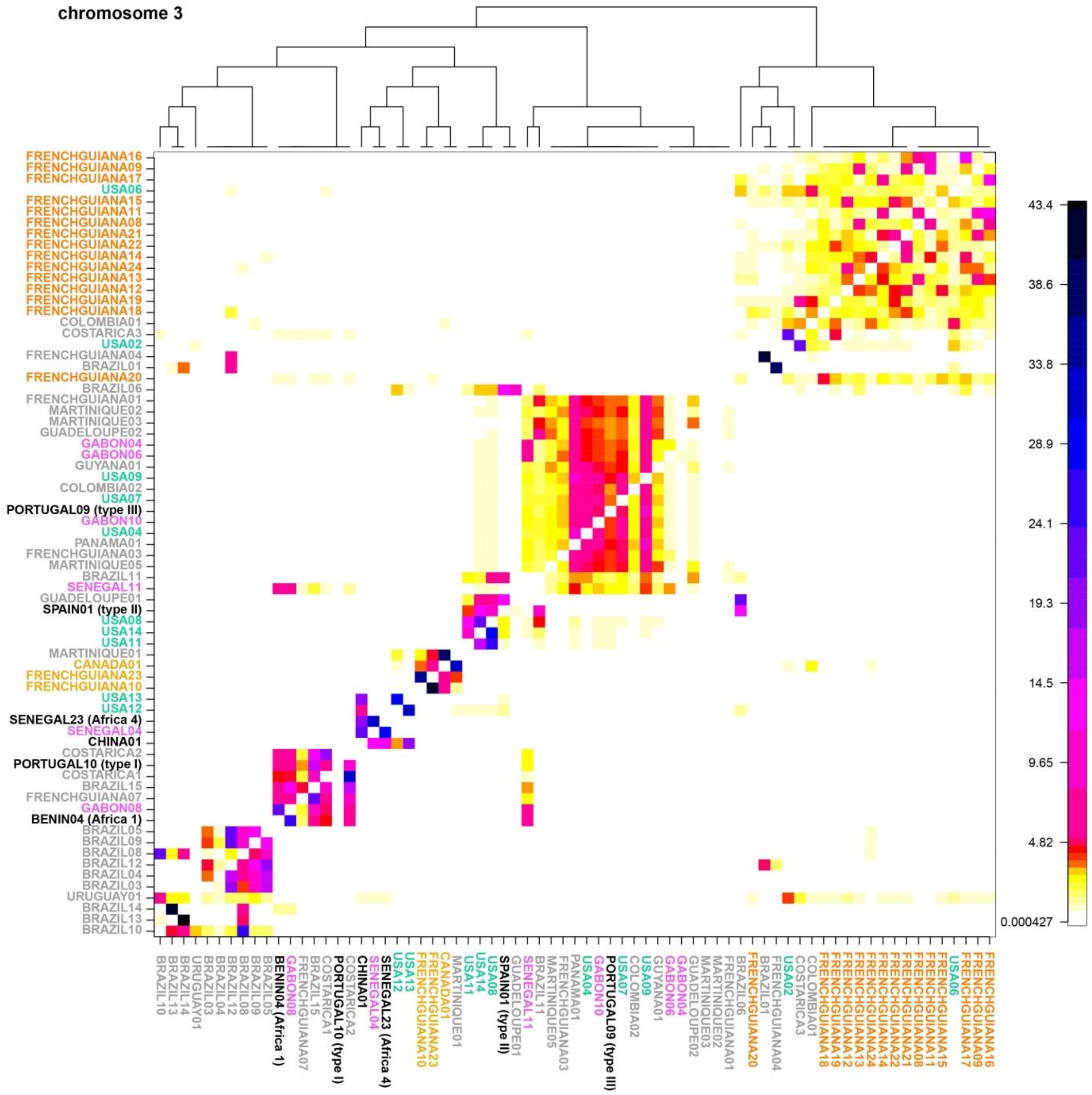

Supplementary Fig. 2e.

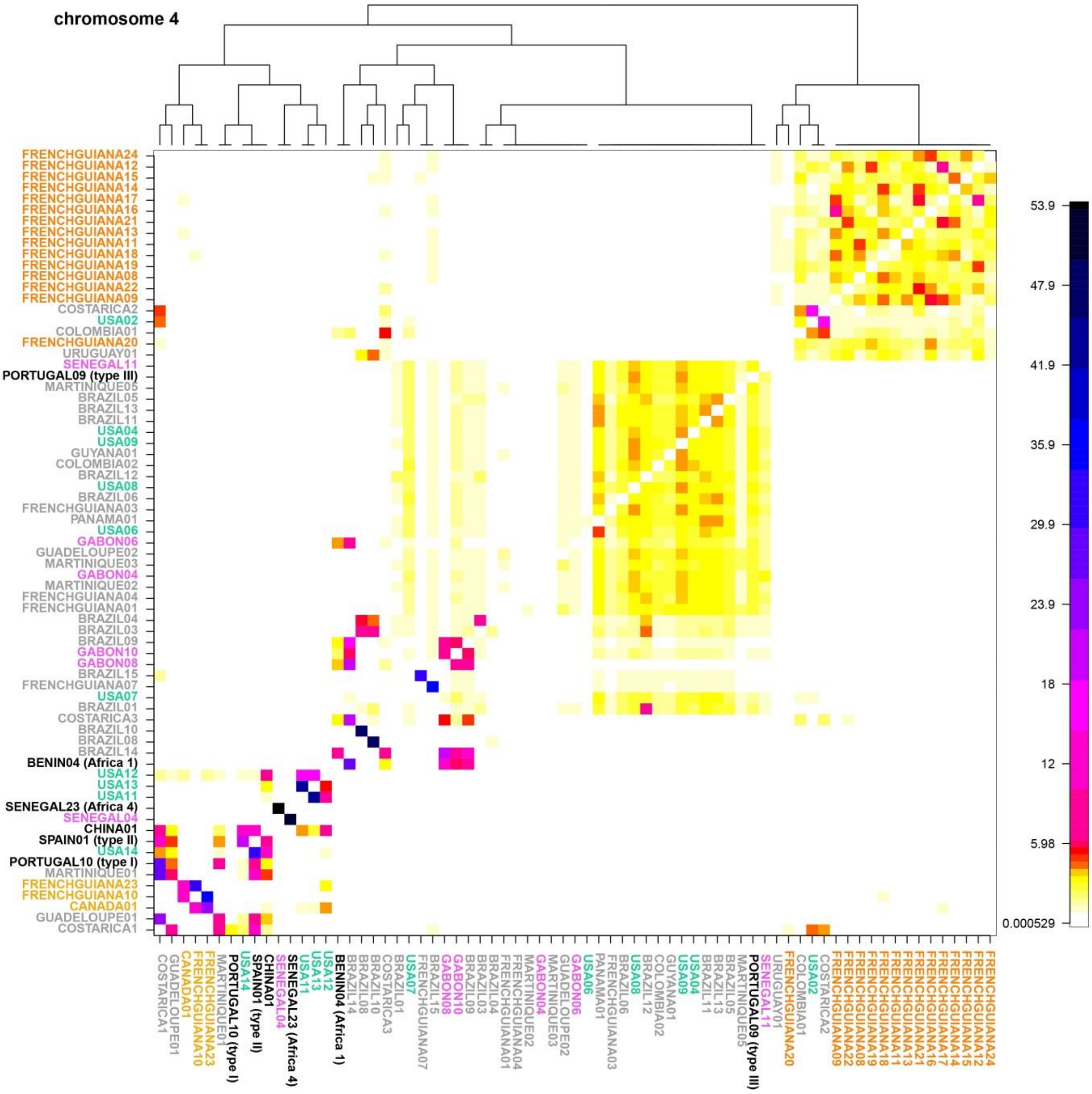

Supplementary Fig. 2f.

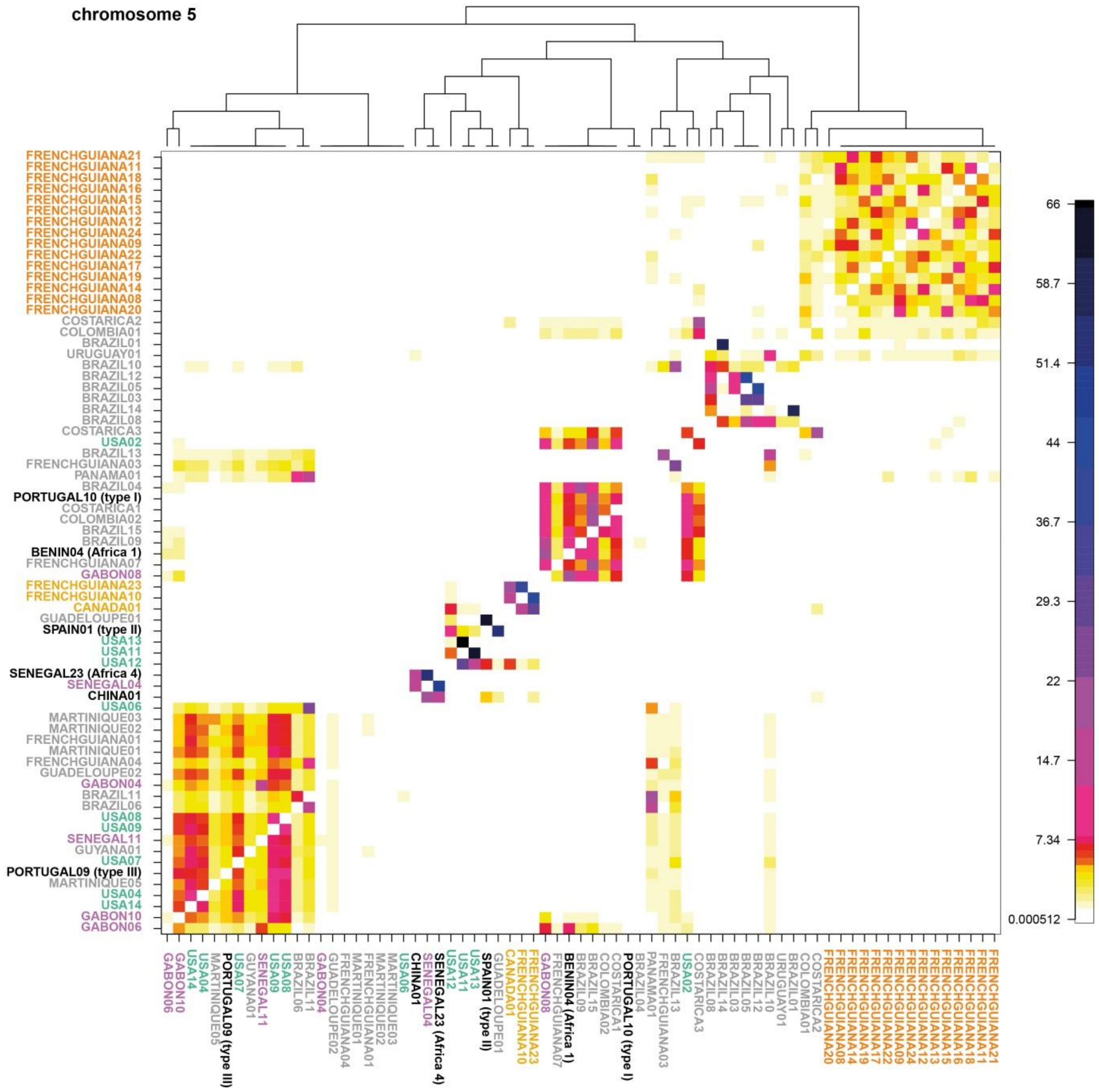

Supplementary Fig. 2g.

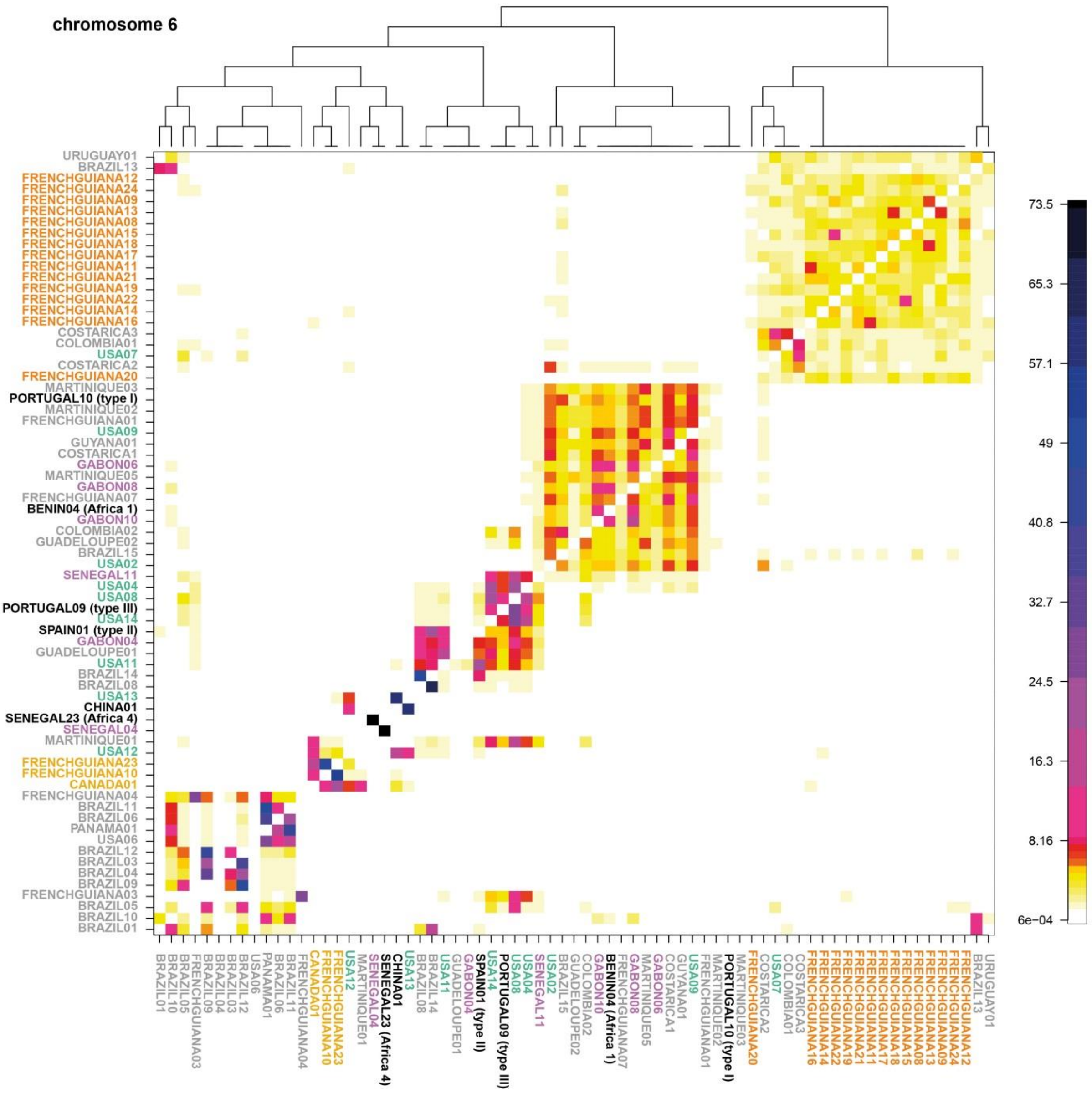

Supplementary Fig. 2h.

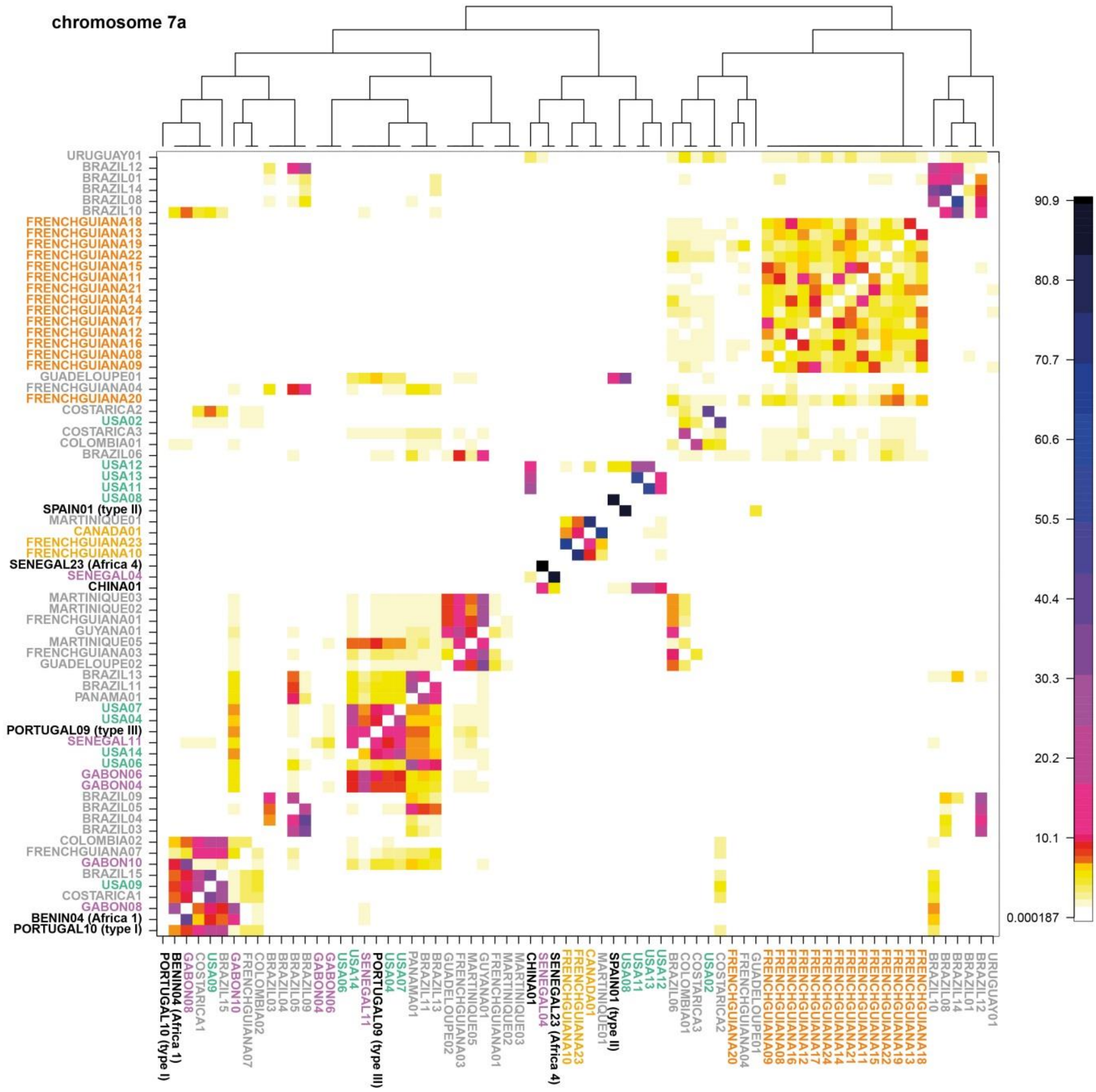

Supplementary Fig. 2i.

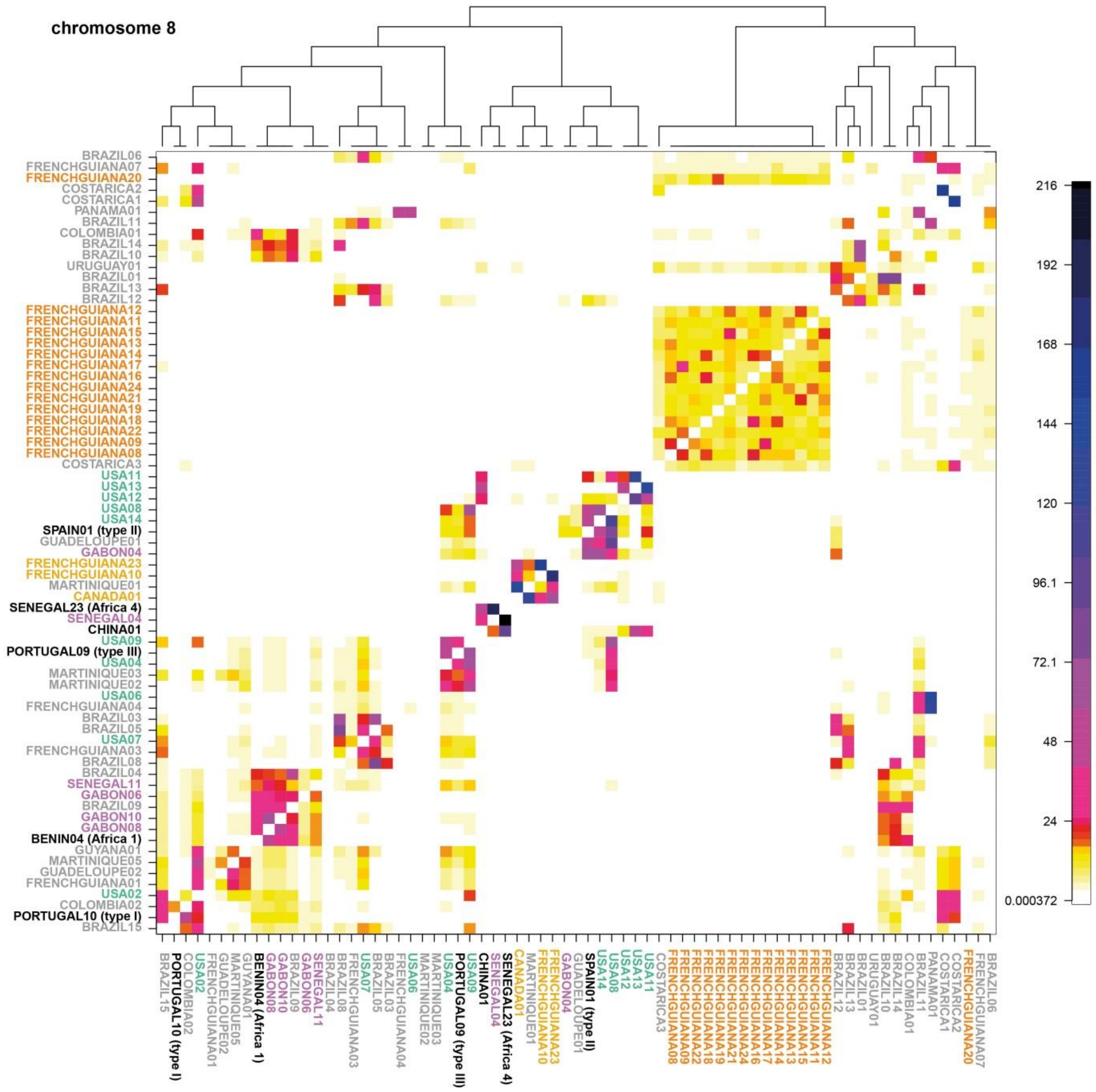

Supplementary Fig. 2j.

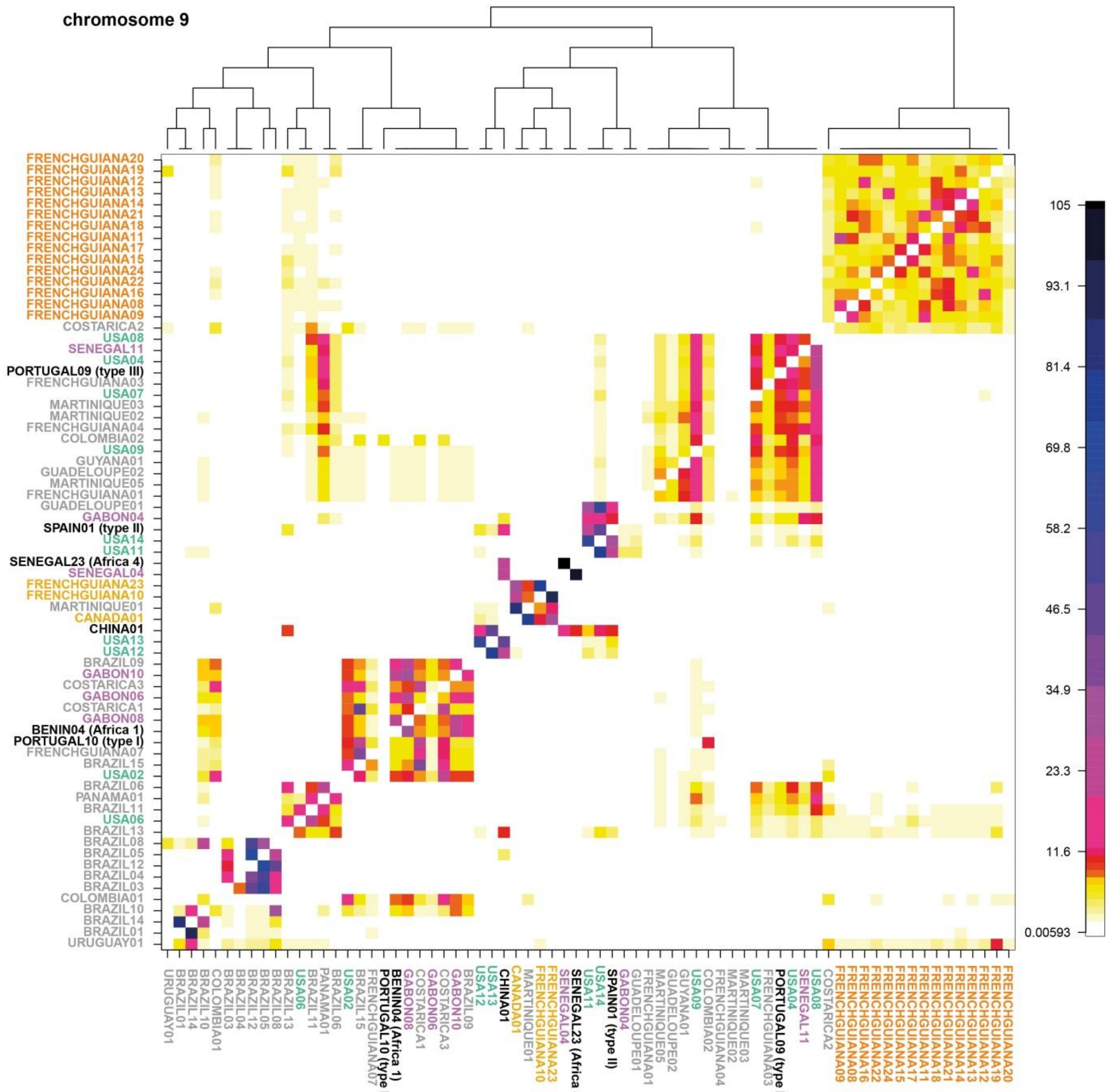

Supplementary Fig. 2k.

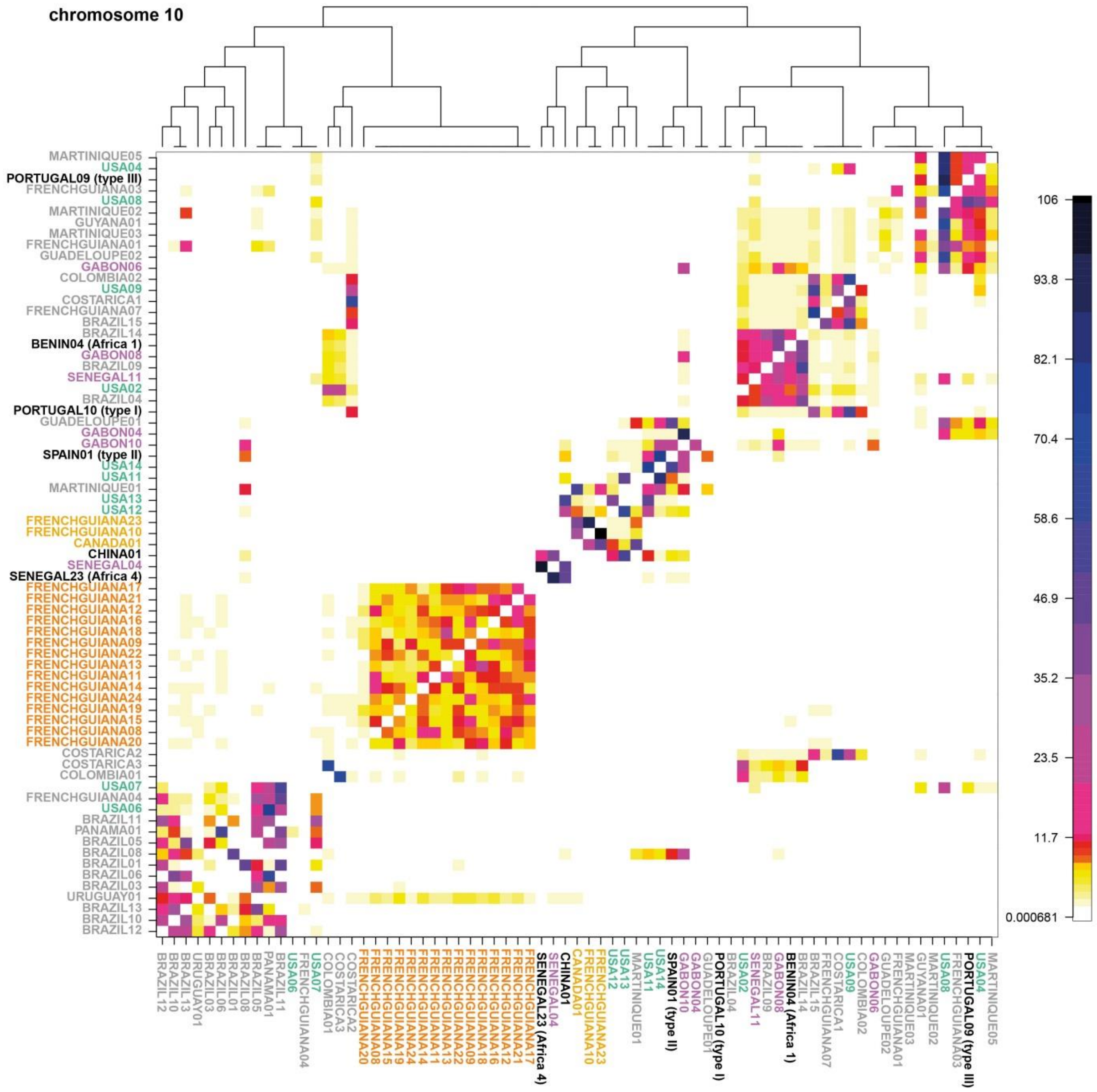

Supplementary Fig. 2l.

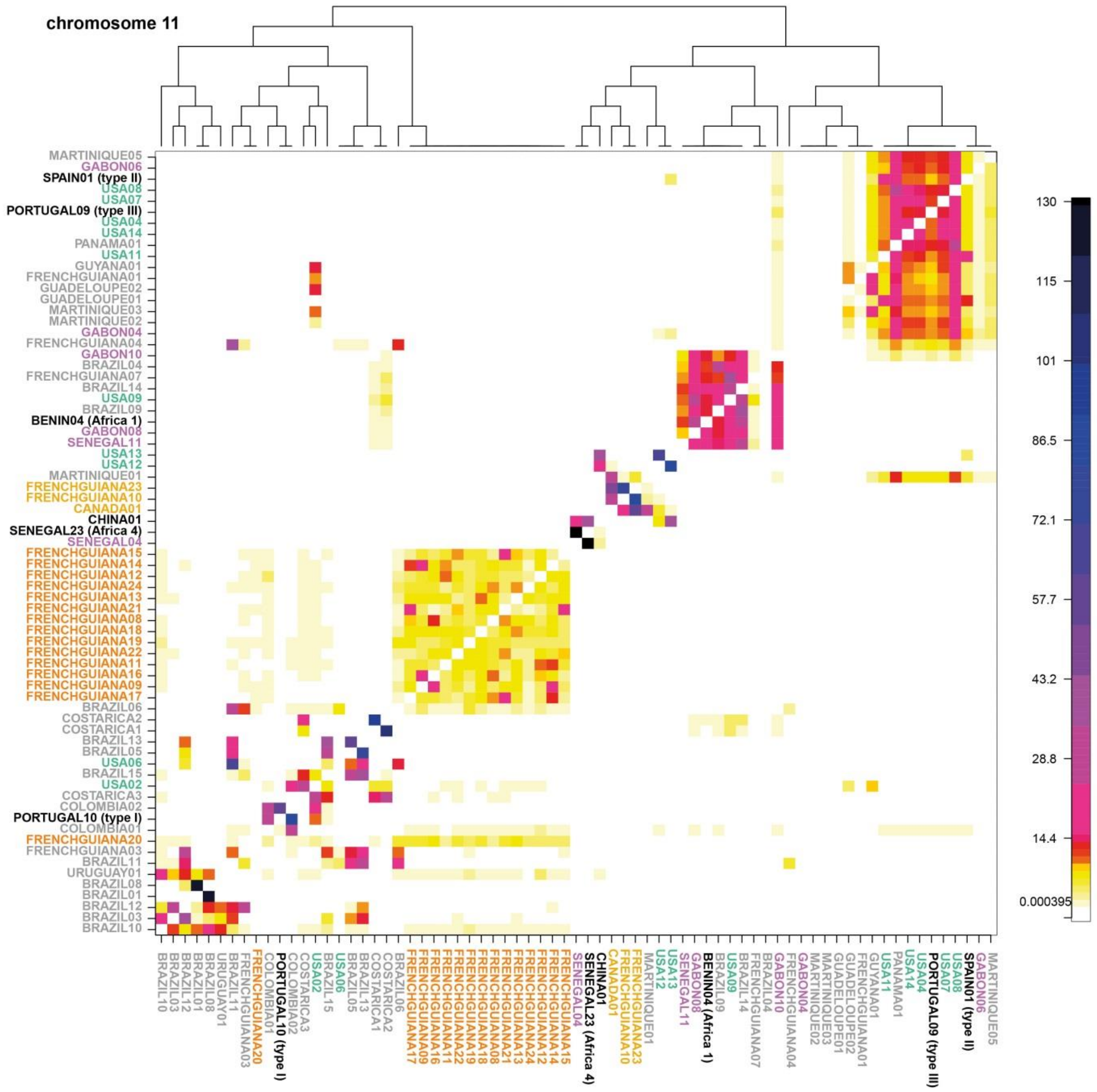

Supplementary Fig. 2m.

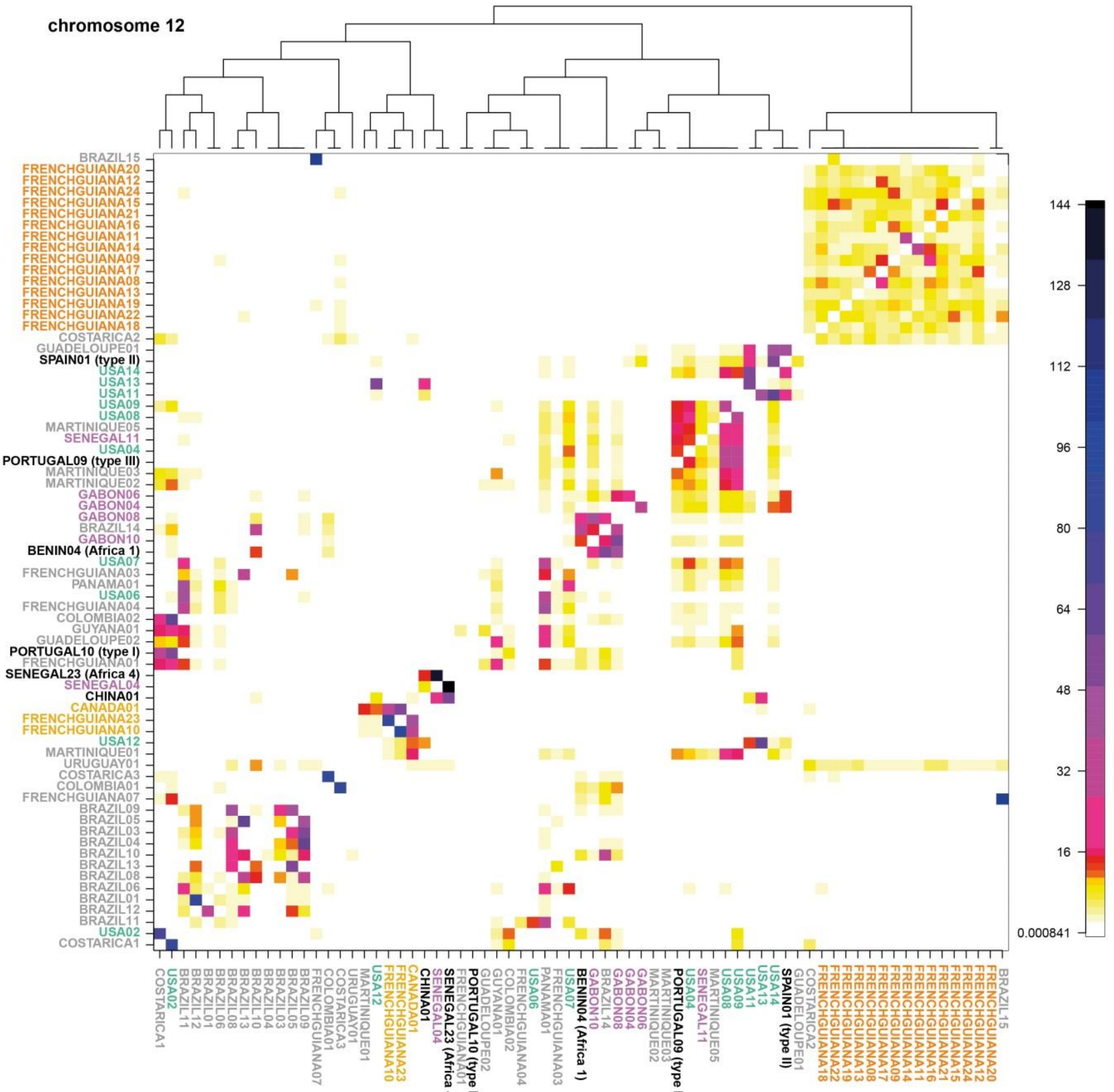

Supplementary Fig. 2n.

Supplementary Fig. 2. ChromoPainter co-ancestry matrices for *Toxoplasma gondii* genomes with population structure assignment based on fineSTRUCTURE analysis. Co-ancestry matrices were generated for the genome-wide dataset of 71 strains and 588,777 SNPs (a), then for each chromosome independently (b-n).The colour of each cell of the matrix indicates the expected number of genetic material (chunks) copied from a donor *T. gondii* genome (x-axis) to a recipient genome (y-axis). On the top is the maximum a posteriori (MAP) tree generated by fineSTRUCTURE which shows the groupings of the different populations. The colour legend of IDs is identical to that of ADMIXTURE plots.

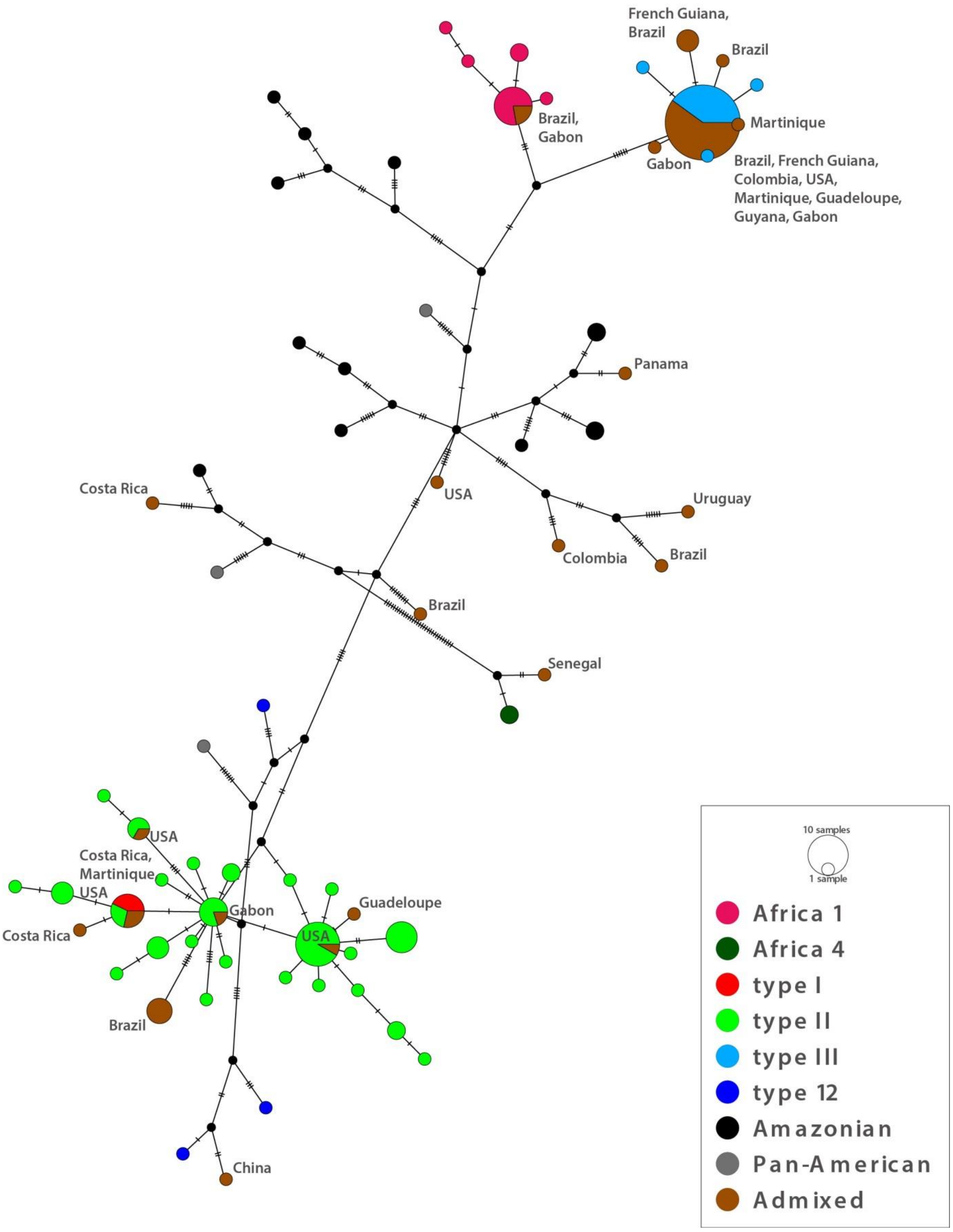

Supplementary Fig. 3. TCS network of apicoplast sequences. Only countries of origin of putative hybrids (in brown) are shown.

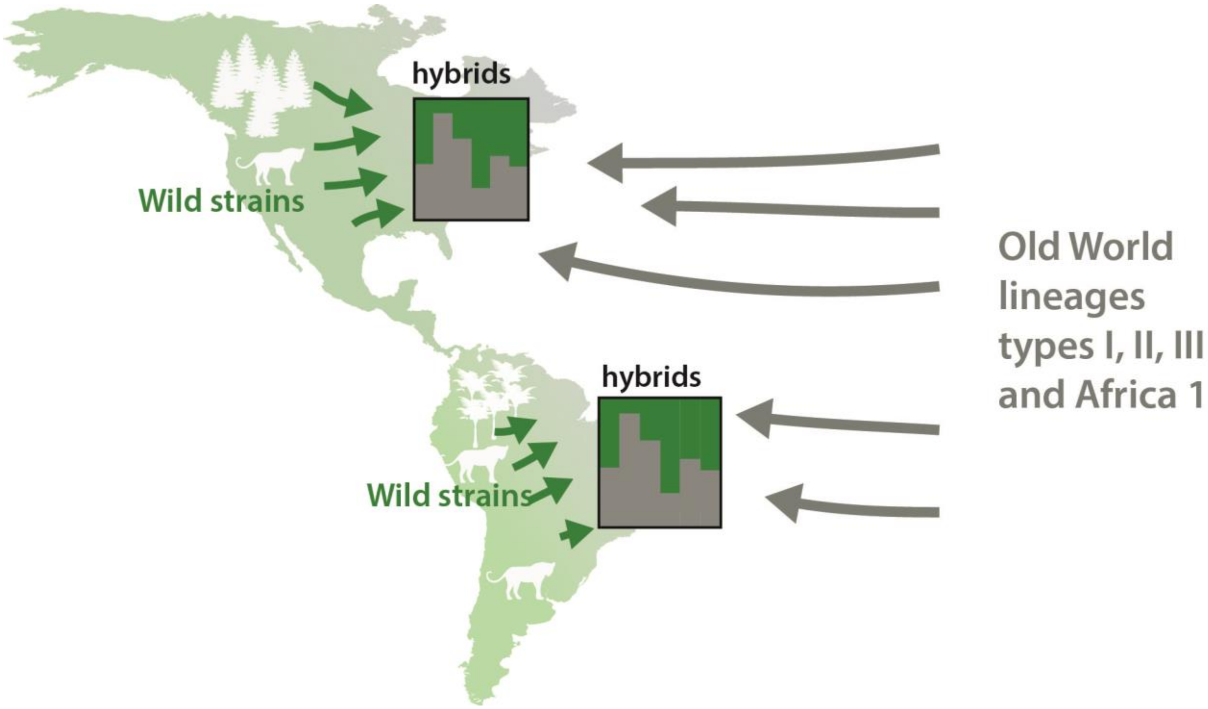

Supplementary Fig. 4. **Figurative illustration of the presumed hybridization process behind emergence of New World hybrid populations of *T. gondii*.**

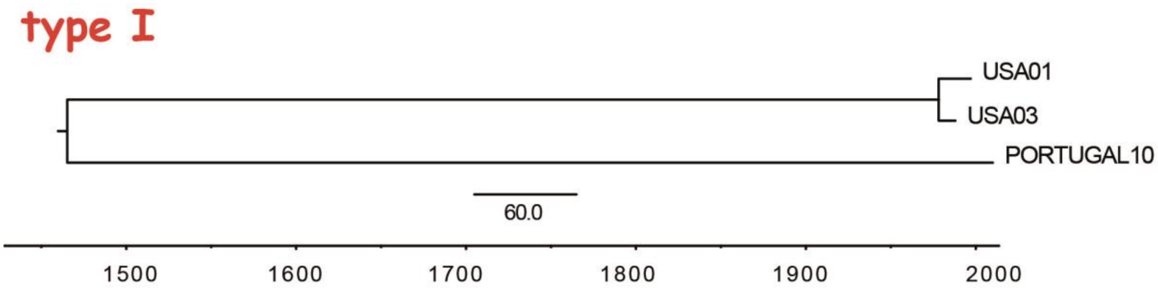
type I
USA01
USA03
PORTUGAL10
60.0
1500
1600
1700
1800
1900
2000

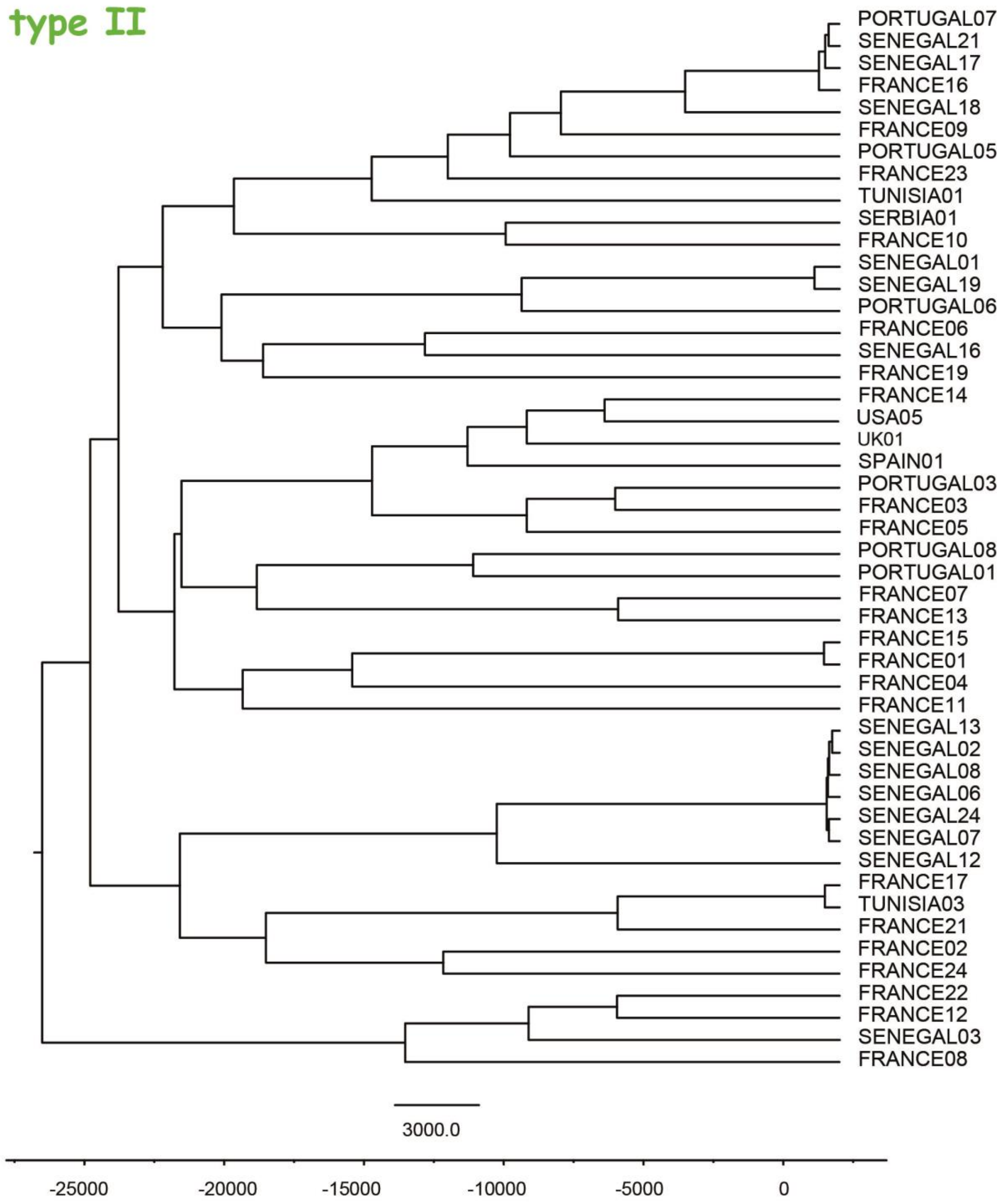
type II
PORTUGAL07
SENEGAL21
SENEGAL17
FRANCE16
SENEGAL18
FRANCE09
PORTUGAL05
FRANCE23
TUNISIA01
SERBIA01
FRANCE10
SENEGAL01
SENEGAL19
PORTUGAL06
FRANCE06
SENEGAL16
FRANCE19
FRANCE14
USA05
UK01
SPAIN01
PORTUGAL03
FRANCE03
FRANCE05
PORTUGAL08
PORTUGAL01
FRANCE07
FRANCE13
FRANCE15
FRANCE01
FRANCE04
FRANCE11
SENEGAL13
SENEGAL02
SENEGAL08
SENEGAL06
SENEGAL24
SENEGAL07
SENEGAL12
FRANCE17
TUNISIA03
FRANCE21
FRANCE02
FRANCE24
FRANCE22
FRANCE12
SENEGAL03
FRANCE08
3000.0
-25000
-20000
-15000
-10000
-5000
0

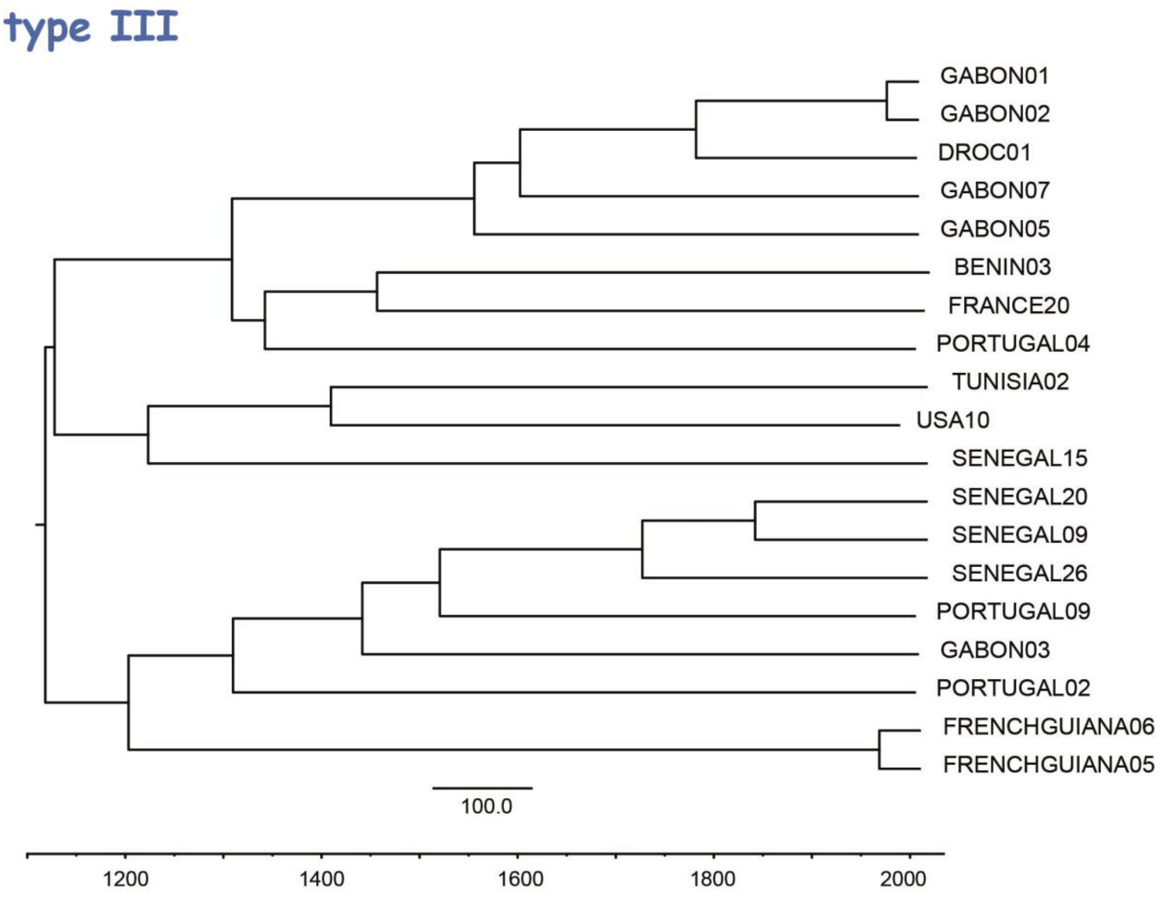

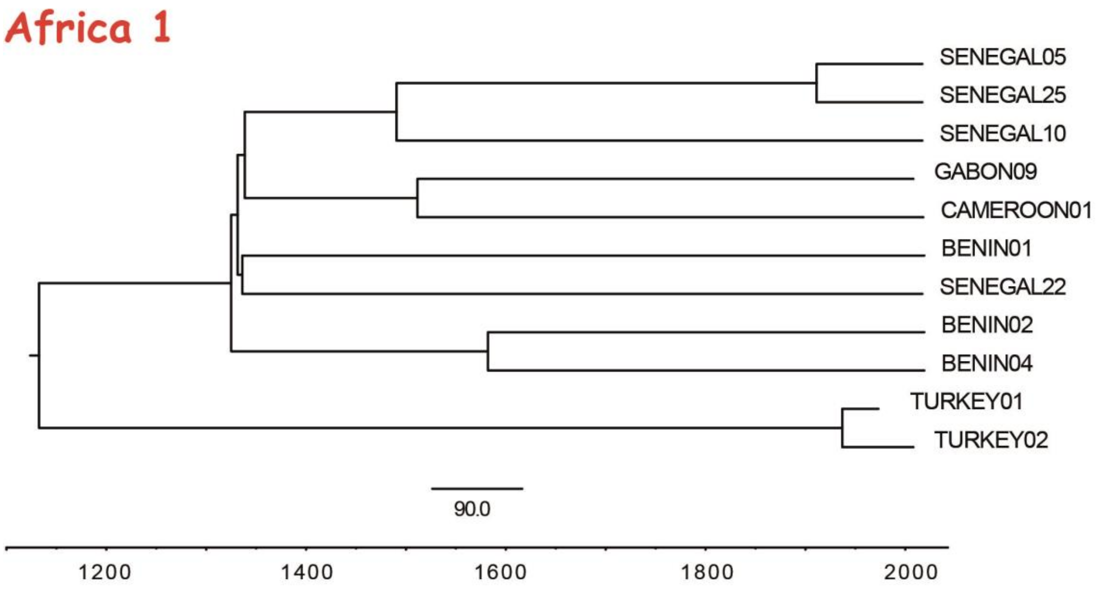

Supplementary Fig. 5. Time-calibrated phylogenetic trees of *Toxoplasma gondii* intercontinental lineages. Bayesian phylogenetic analyses were carried out to estimate the time to the most recent common ancestor (TMRCA) of each of the four major intercontinental clonal lineages and the divergence times between Old World and New World strains of the same lineage.

chromosome 1a

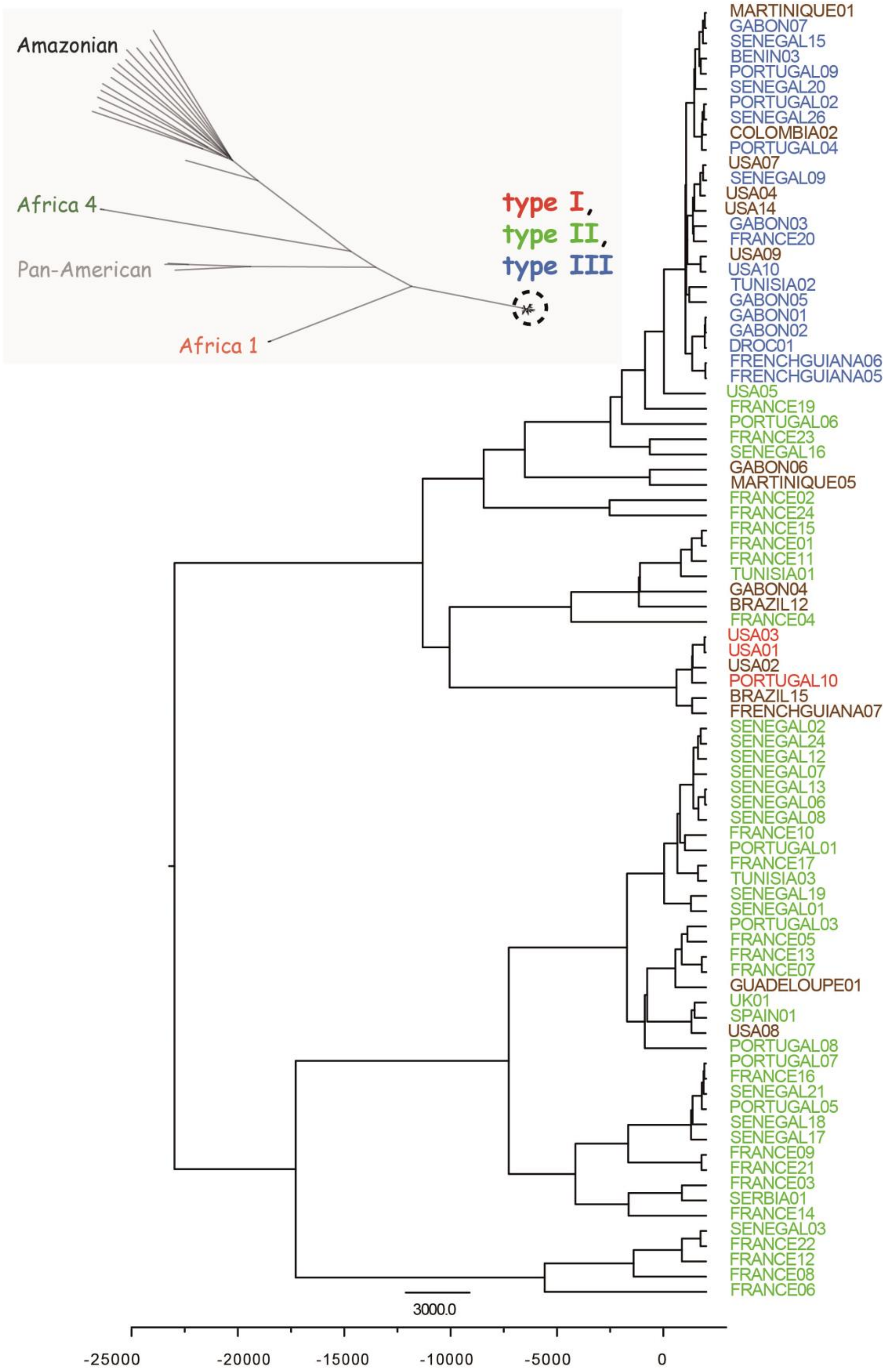

Supplementary Fig. 6a

chromosome 1b

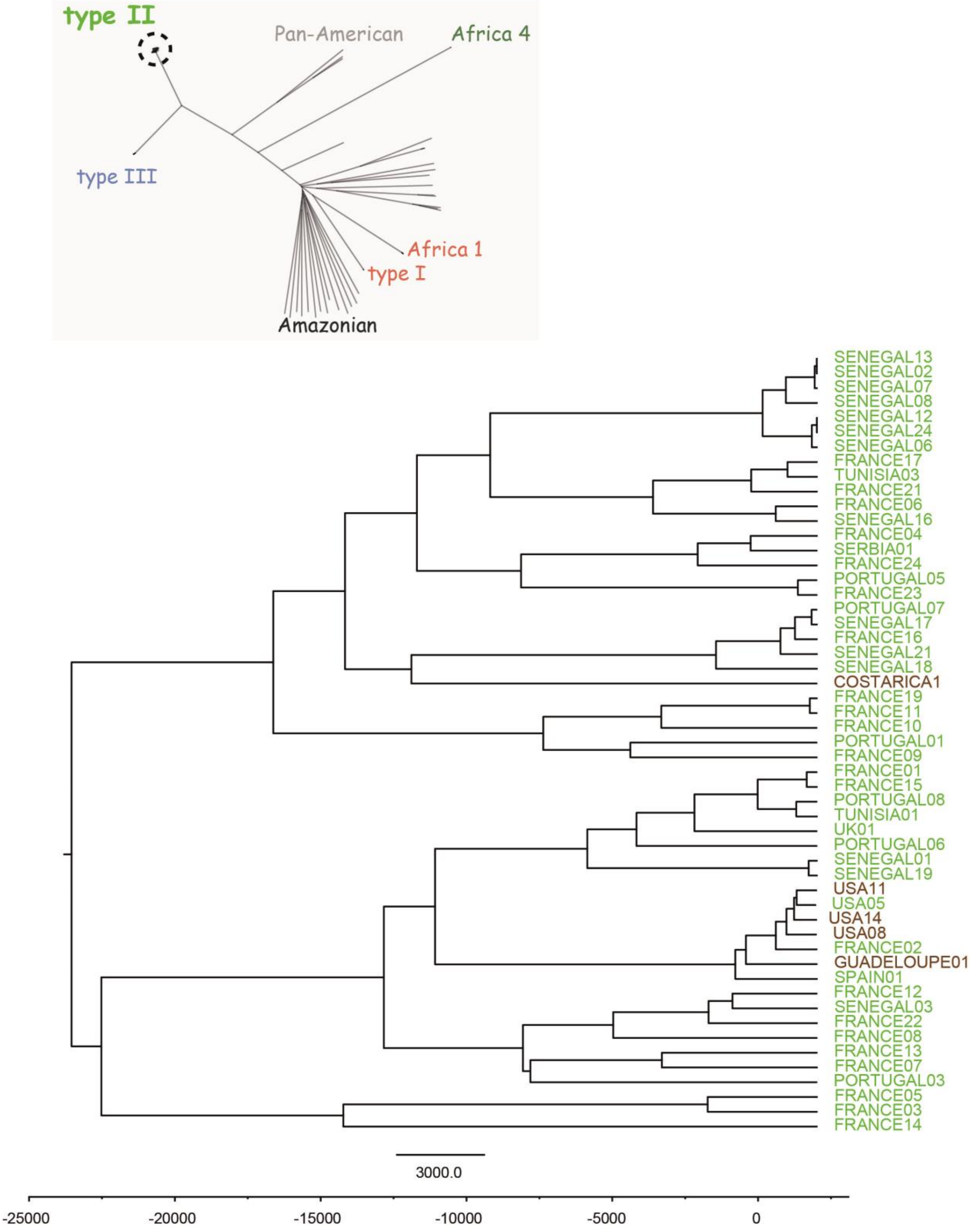

Supplementary Fig. 6b

chromosome 1b

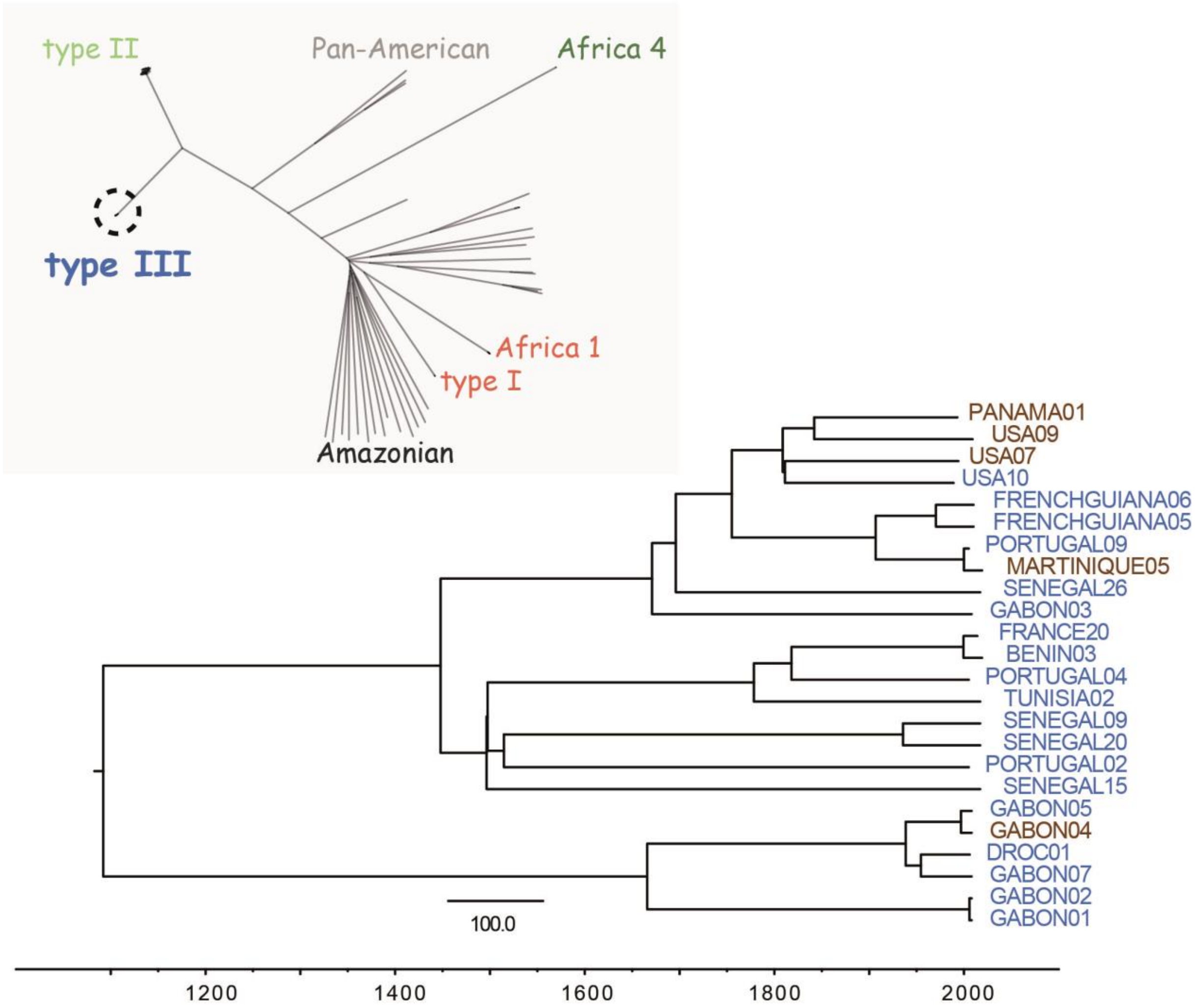

Supplementary Fig. 6c

chromosome 2

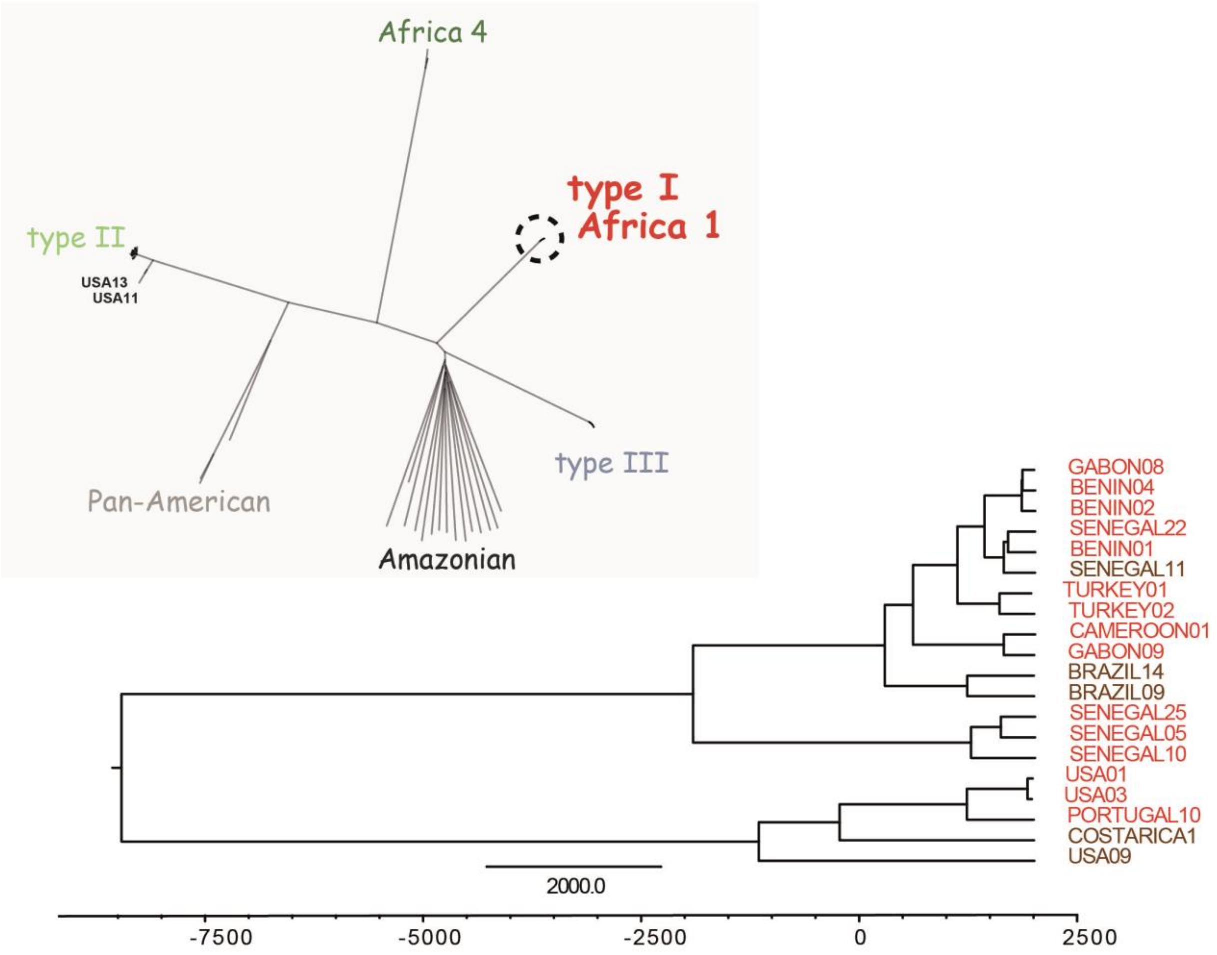

Supplementary Fig. 6d

chromosome 2

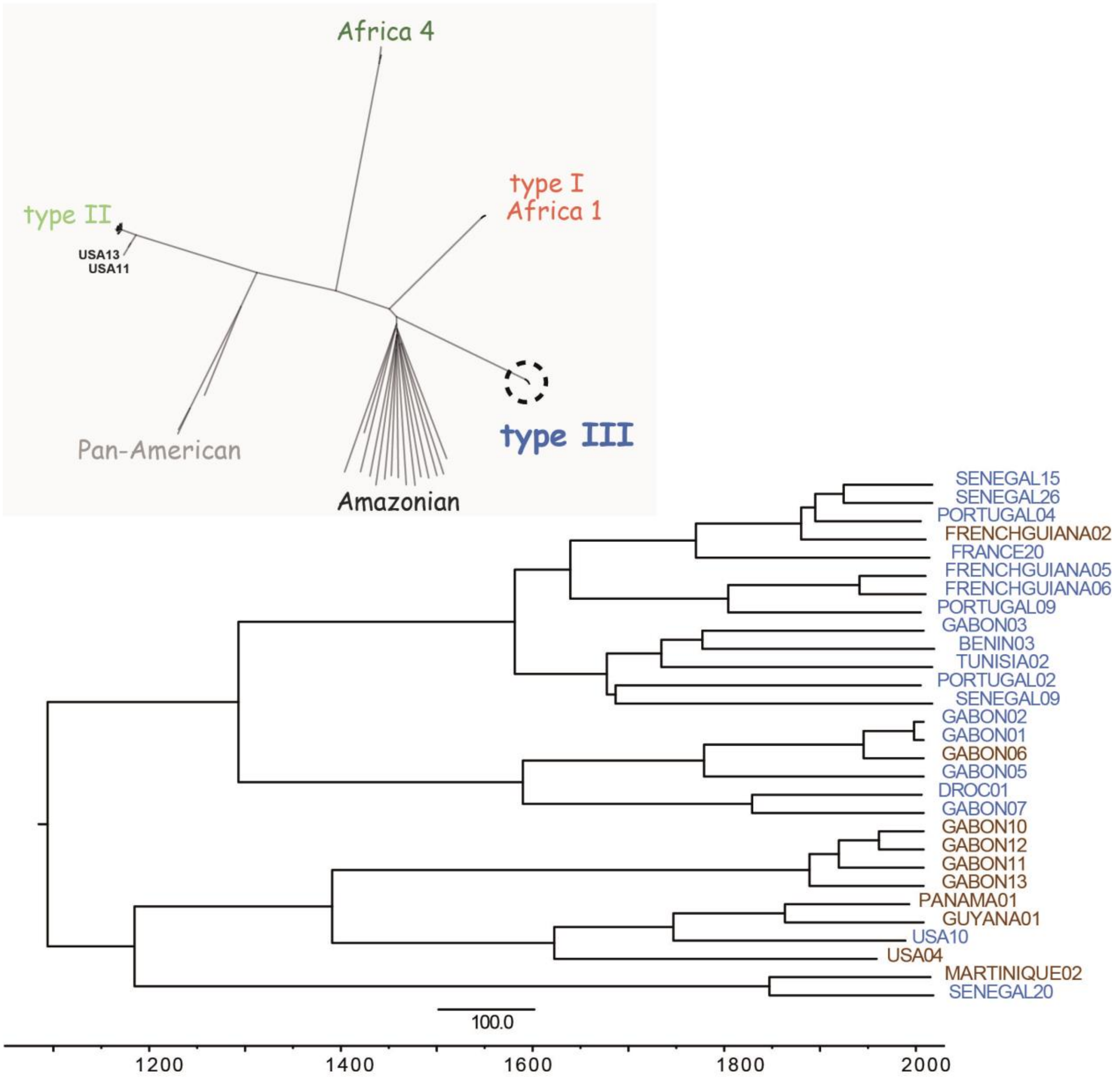

Supplementary Fig. 6e

chromosome 3

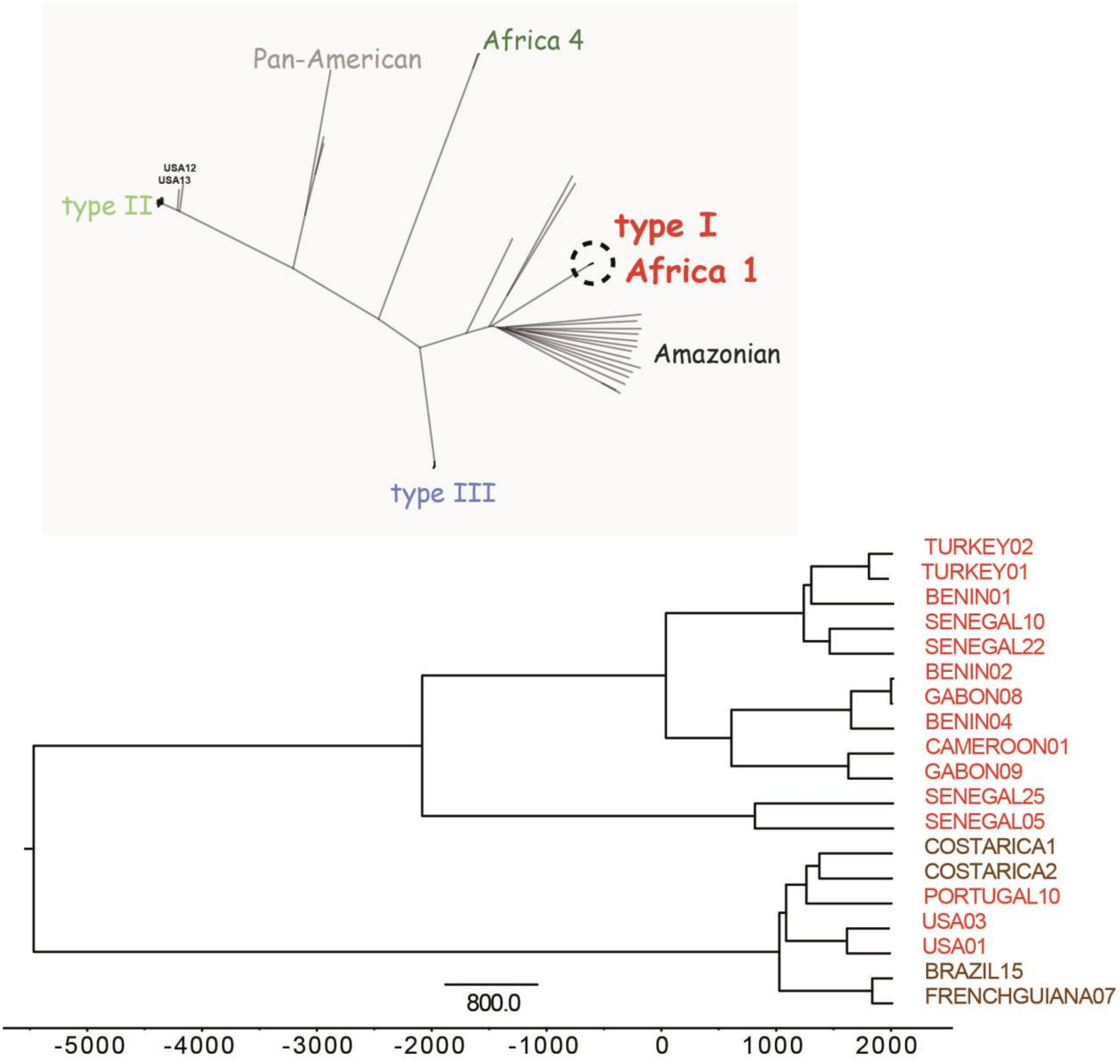

Supplementary Fig. 6f

chromosome 3

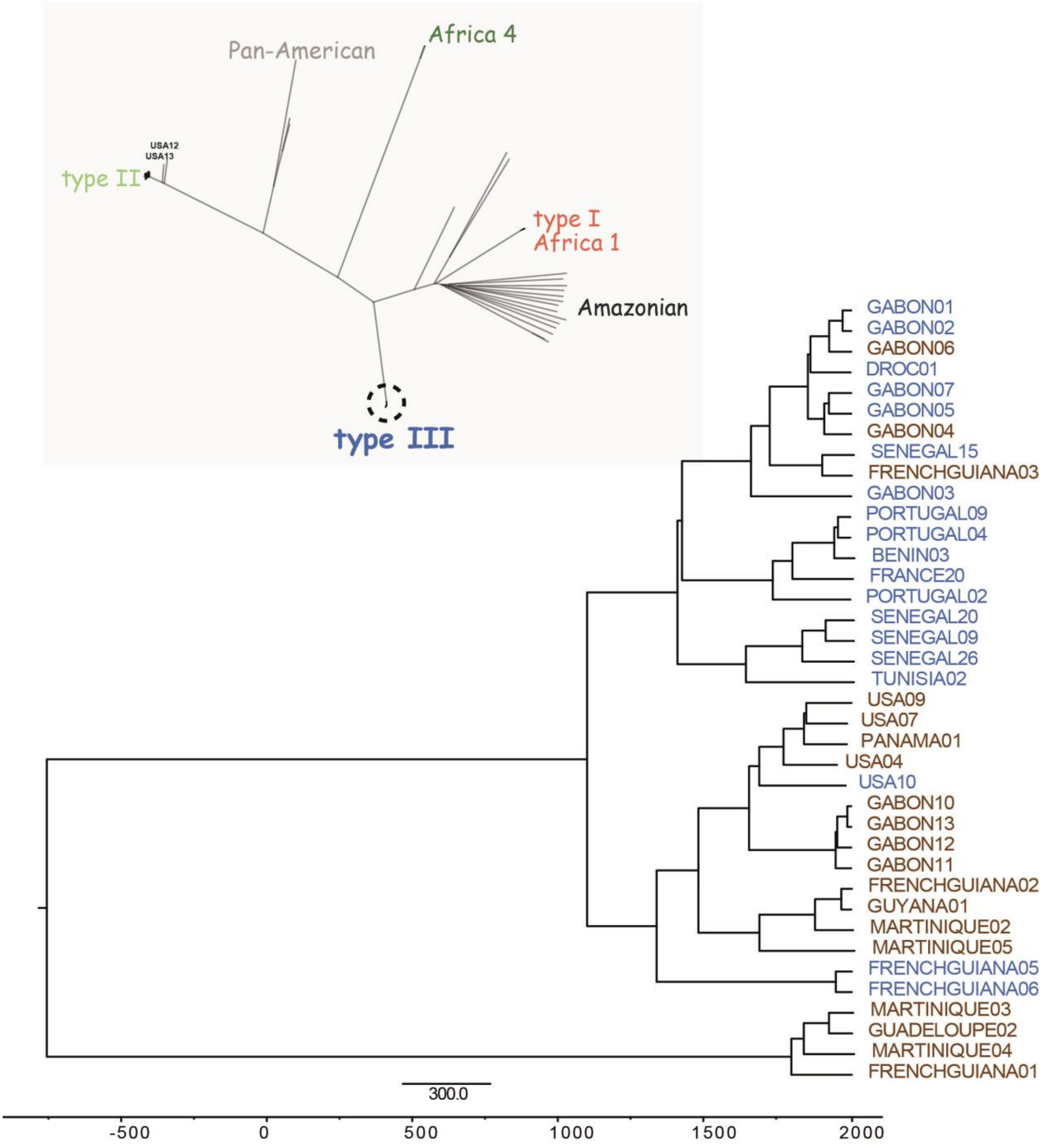

Supplementary Fig. 6g

chromosome 4

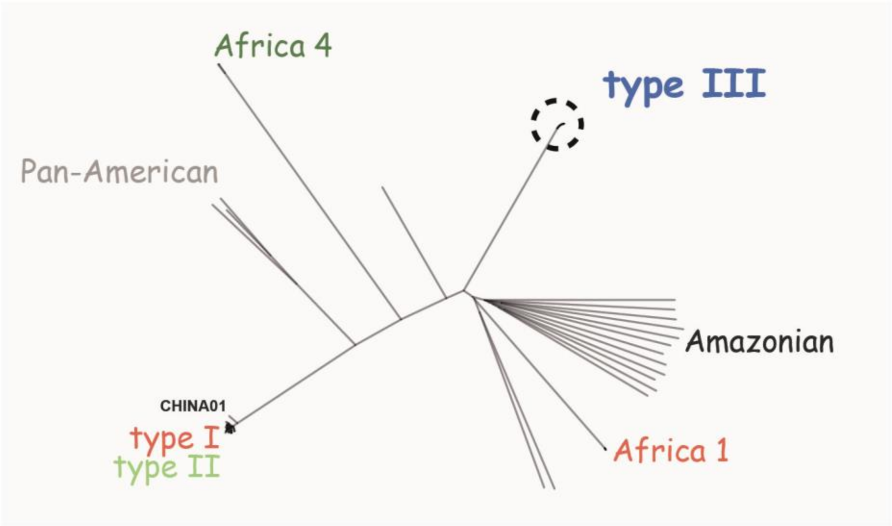

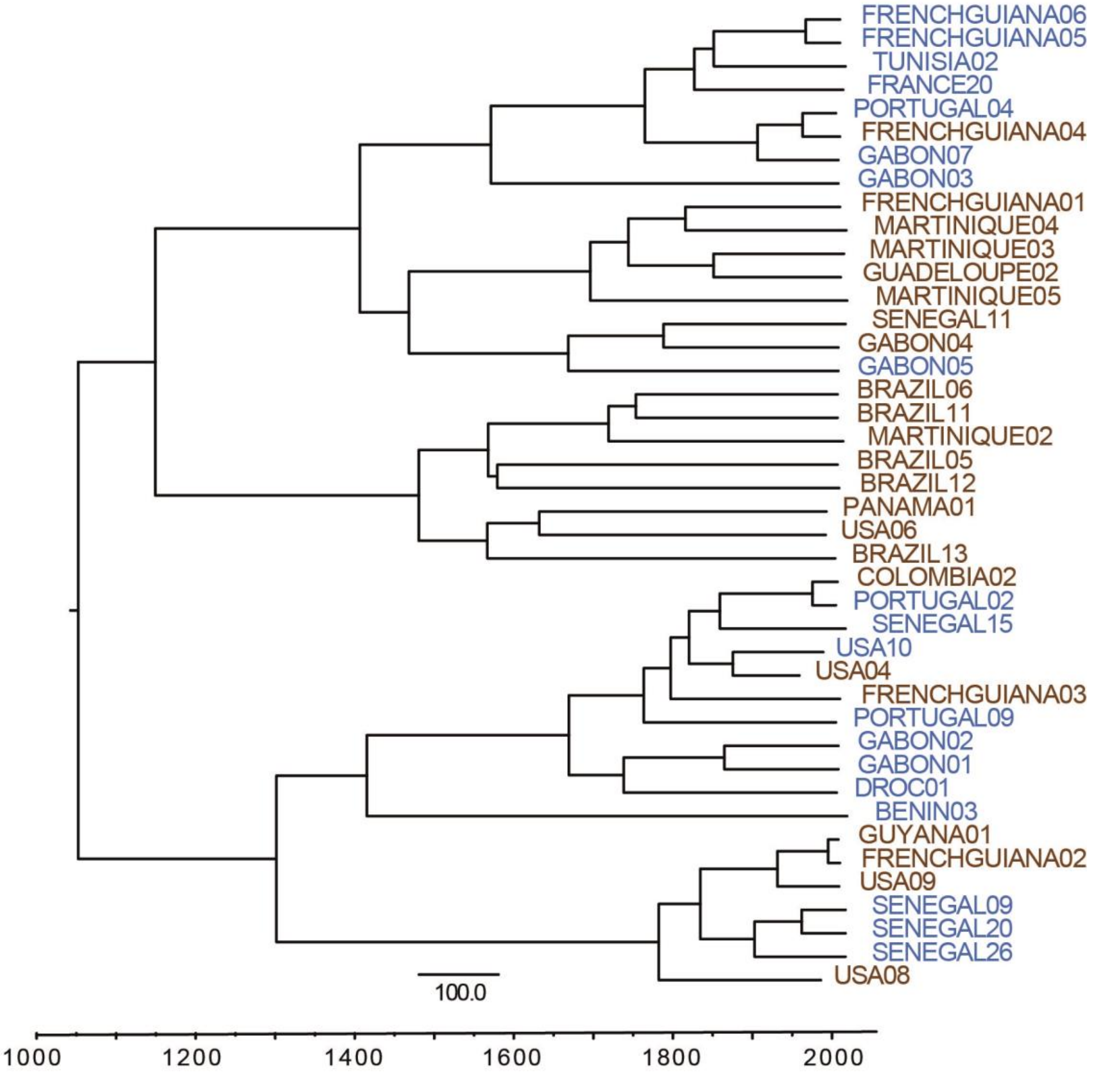

Supplementary Fig. 6h

chromosome 5

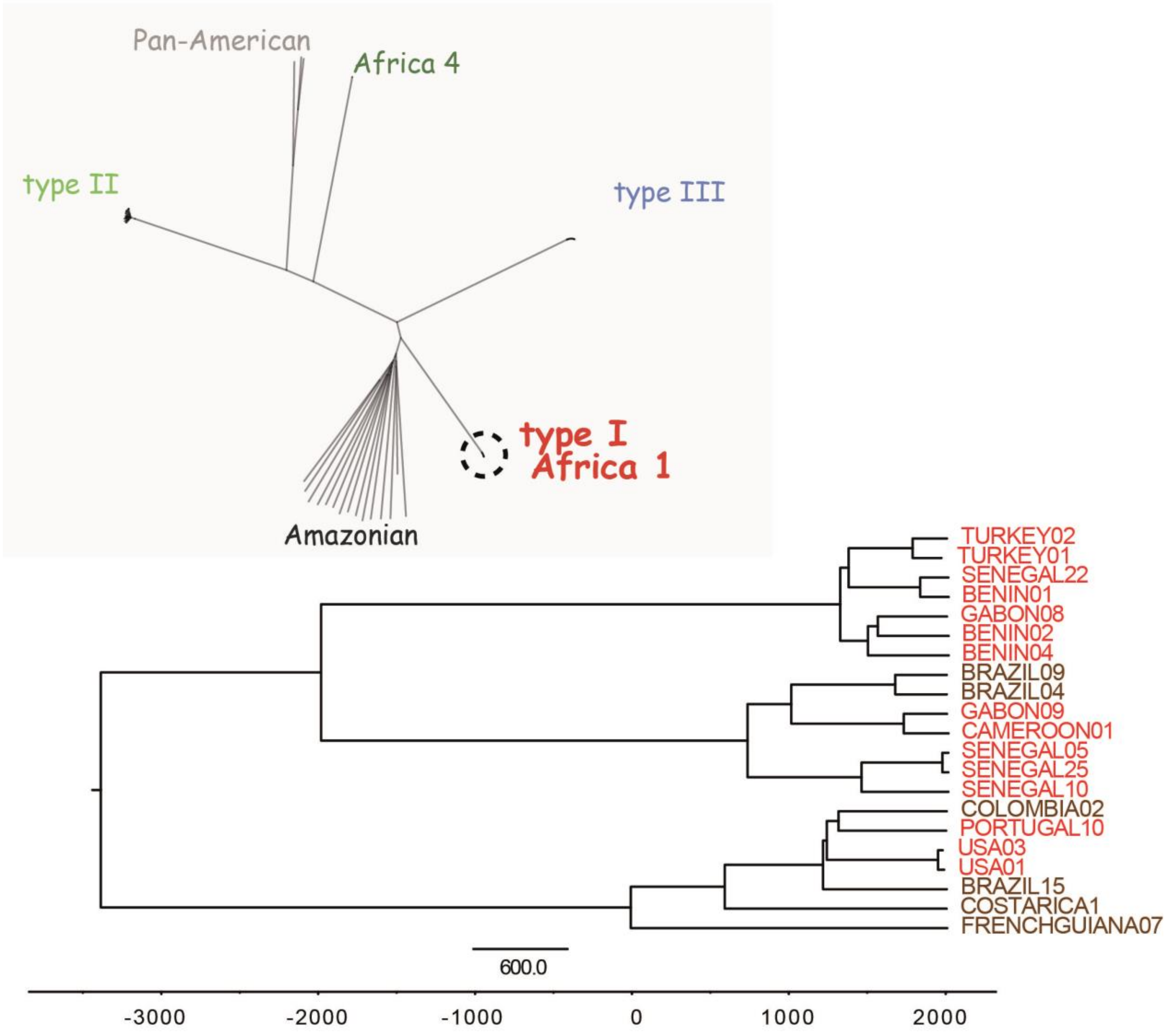

Supplementary Fig. 6i

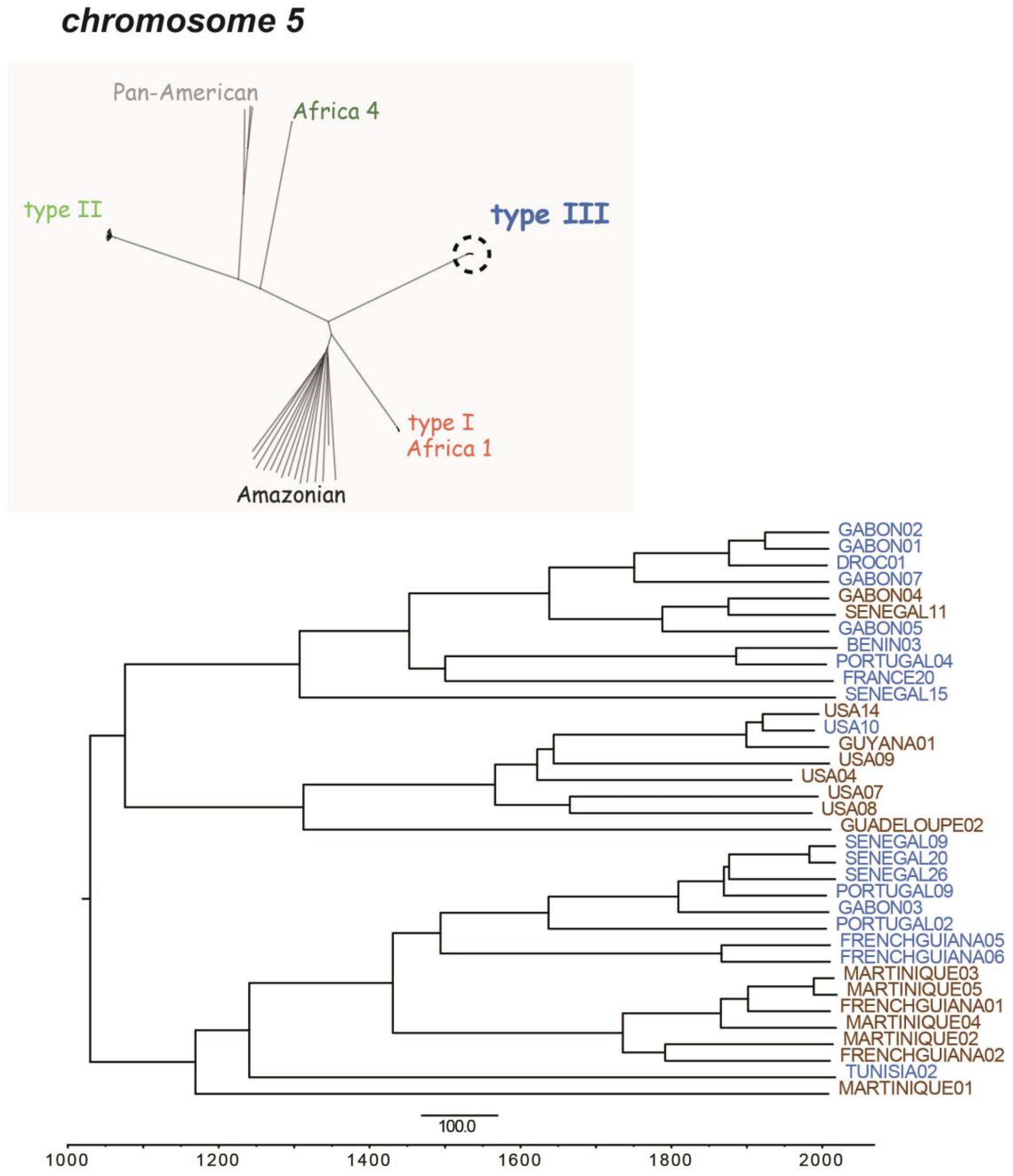

Supplementary Fig. 6j

chromosome 6

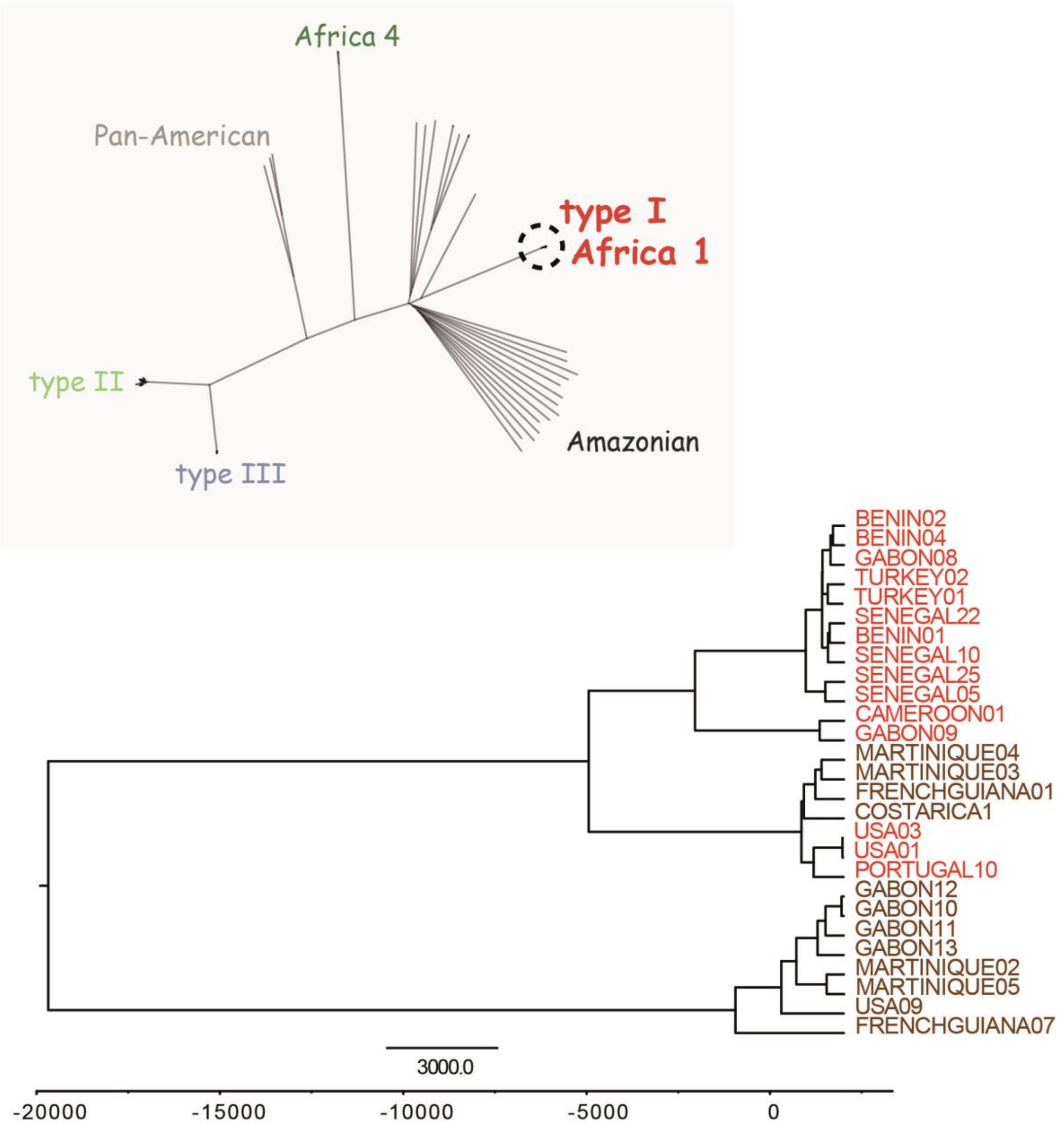

Supplementary Fig. 6k

chromosome 7a

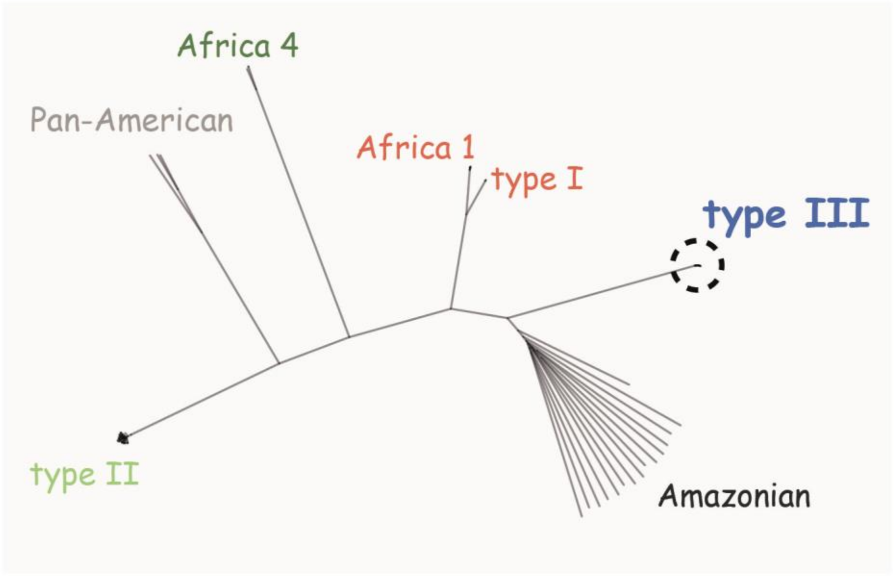

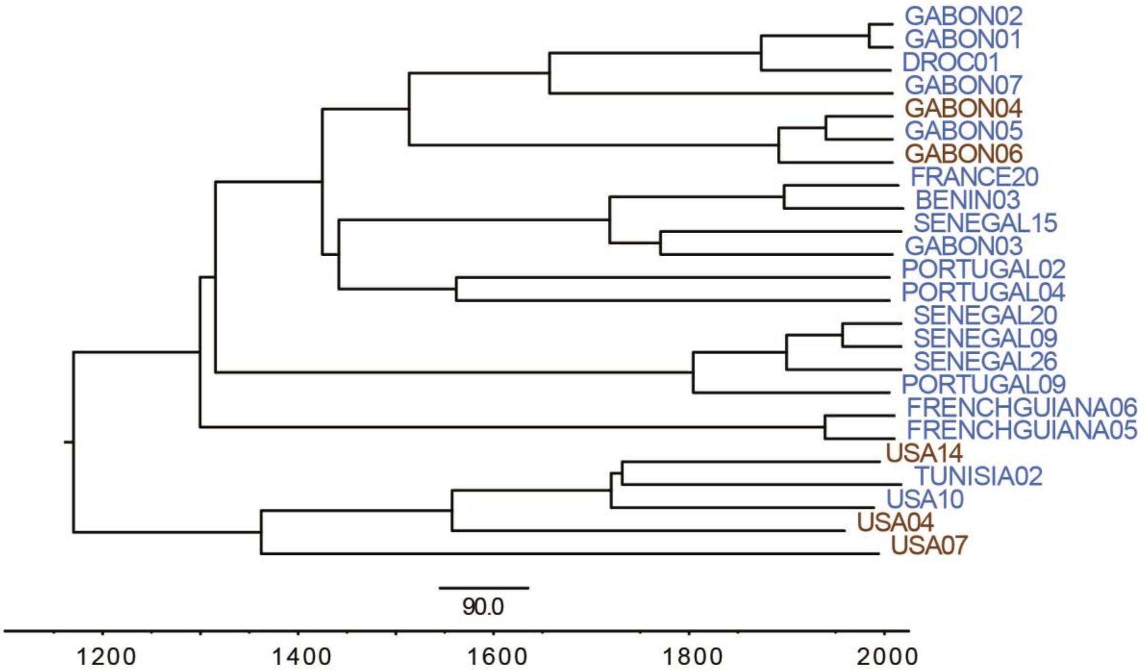

Supplementary Fig. 6l

chromosome 11

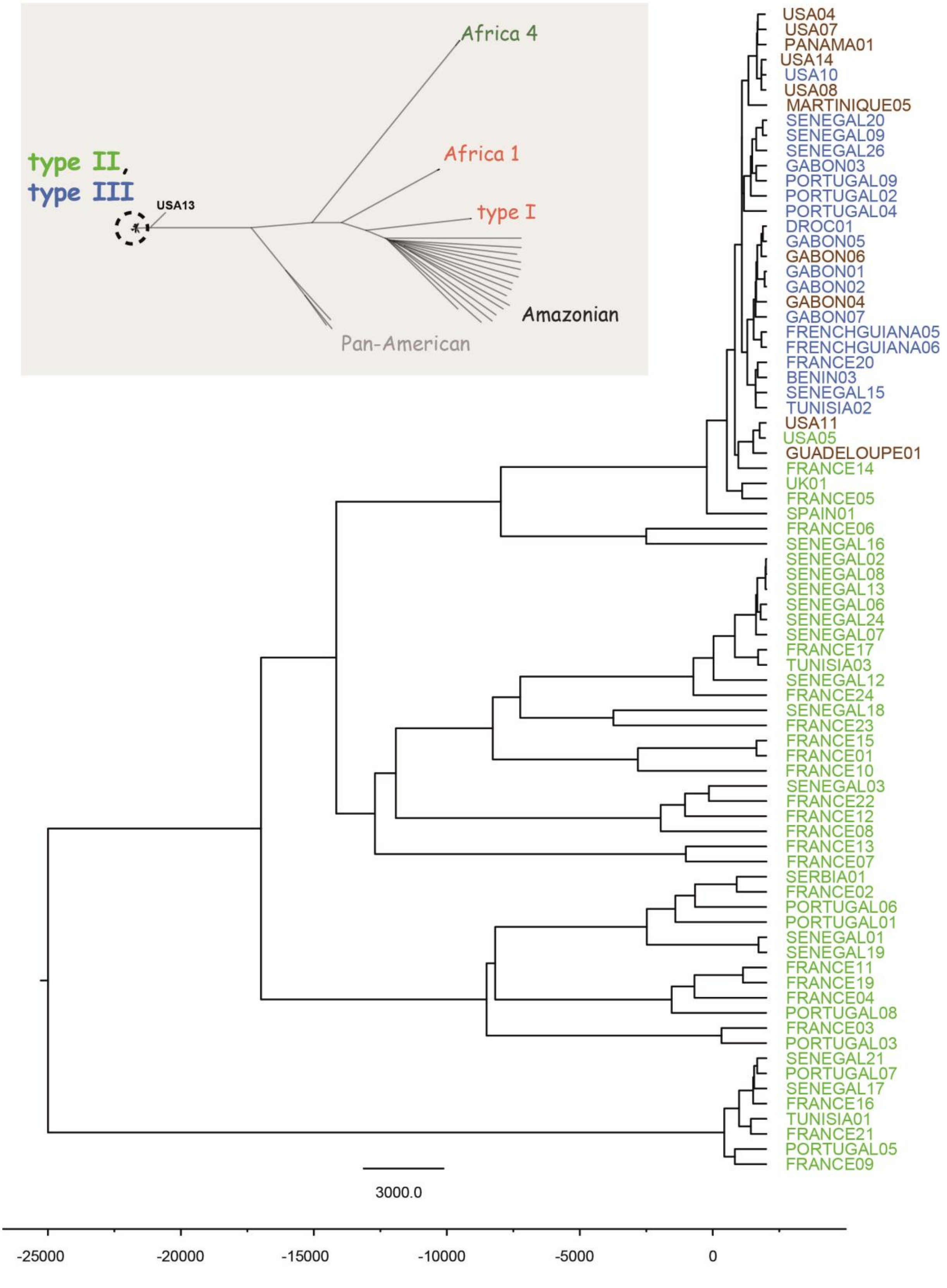

Supplementary Fig. 6m

Supplementary Fig. 6. Genetic divergence and time-calibrated phylogenies of *Toxoplasma gondii* Old World and New World populations at chromosome level. When at least five putative hybrid strains (refer to Fig. 3) carried a complete chromosome of ancestry similar to that of one of the intercontinental lineage (either type I, type II, type III or Africa 1), a neigbour-joining (NJ) tree was produced for this specific chromosome (on gray background) by including chromosome sequences of all ancestral populations and putative hybrid strains having a unique (non-admixed) ancestry at this chromosome. Time-calibrated phylogenetic trees were produced with BEAST from chromosome sequences exhibiting no obvious divergence on NJ trees (branches surrounded by a dotted circle). Tips labels of type I and Africa 1 strains are represented in red, type II strains in green, type III strains in blue and putative hybrids in brown.

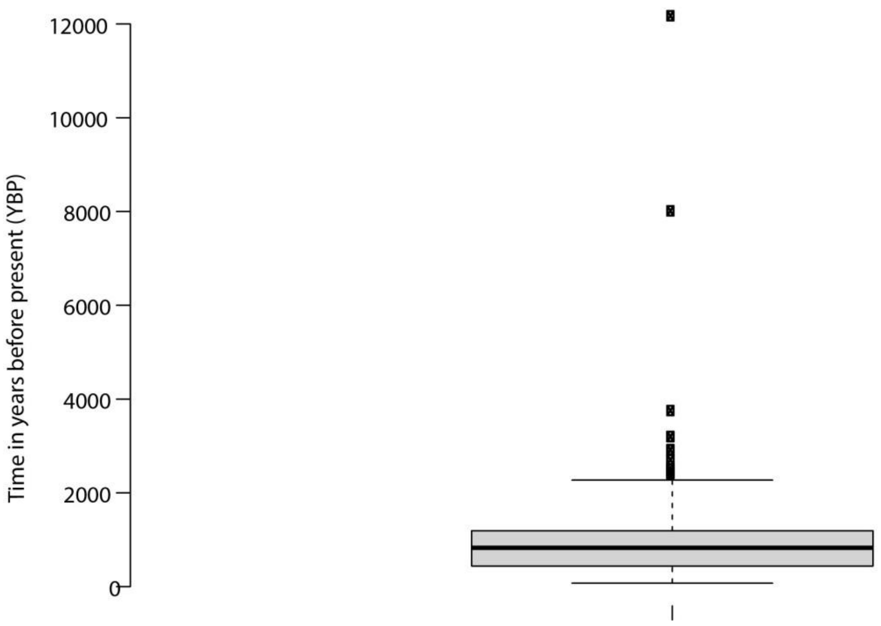

Supplementary Fig. 7. Divergence times between Old and New World strains. The mean time to the most recent common ancestor TMRCA of each New World strain with its closest Old World relative was extracted from each phylogenetic tree generated with BEAST (Supplementary Fig. 5-6). A boxplot of these mean TMRCA (n=112) was produced showing a median value of 827, an interquartile range of 759 (439-1,198), a lower and upper whiskers of 73 and 2,272, respectively, a minimum of 73 and a maximum of 12,175.

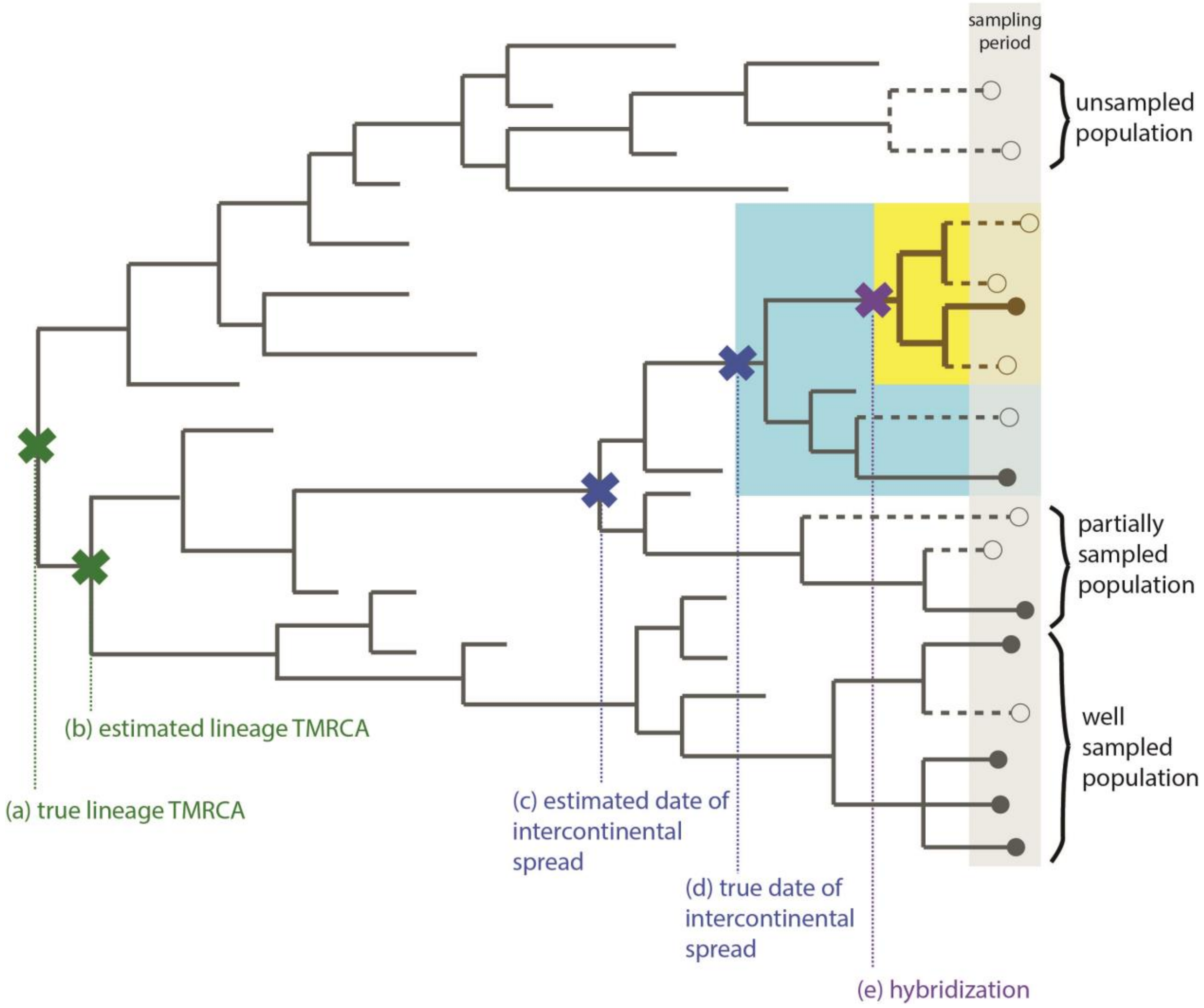

Supplementary Fig. 8. Figurative illustration of sampling and methodological biases in dating estimates. In order to obtain the most accurate estimation of a lineage emergence time (a), strong sampling of most current populations within the lineage is mandatory and omitting certain populations (sublineages) within the lineage are likely to lead to an underestimation of the lineage emergence time (b). It is also noteworthy that the estimated lineage TMRCA from sampling of current populations could also underestimate the true lineage emergence time if certain populations have collapsed during the lineage existence period. Approximations of intercontinental migration times were obtained from TMRCA of the most recent nodes where Old World and New World branches coalesce in each phylogenetic tree. However, these TMRCA estimates correspond to divergence times between Old World and New World branches (c). Divergence is likely to have existed before the time of migration (d). In addition, probable source populations (*T. gondii* populations that spread between Old and New Worlds) were only partially sampled in this study since several port cities could not be sampled (e.g. Liverpool, Loango, Lisbona, Nantes). Isolates not belonging to source populations are expected to exhibit some degree of divergence from the source populations, and consequently from individuals that have undergone intercontinental spread from these source populations. This divergence bias is therefore probably resulting in an overestimation of migration times. Optimally, sampling should include isolates from all port areas historically involved in transatlantic and colonial trade in Europe and Africa, which is only partially the case in this study. In consistence with the model developed in this study, migration times between Old and New Worlds

are expected to have occurred within the last 500 years. Propagules (individuals that have undergone intercontinental spread) and propagules progeny are represented on a blue background. Many strains share large genomic portions (including complete chromosomes) with the major intercontinental lineages. These strains are probably recent hybrids of at least one of these lineages (e). The genomic portions (e.g. a complete chromosome) inherited from one of these lineages continue to evolve and gradually diverge from their parental lineages. They are represented by brown tranches on a yellow background.

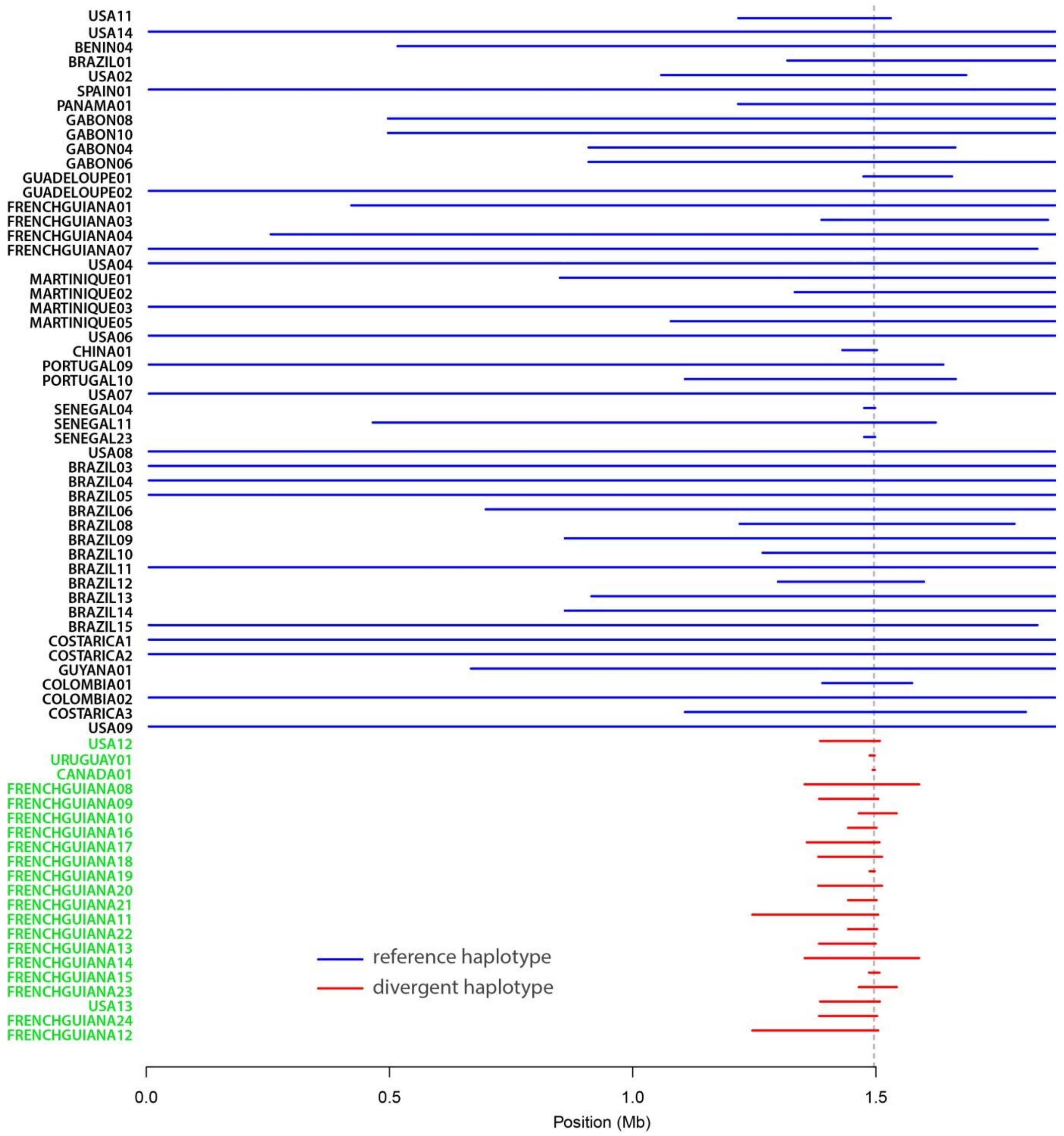

Supplementary Fig. 9. Linkage disequilibrium around the outlier region of selection. Haplotype length around the position 1495970 on chromosome 1a in *T. gondii* strains carrying the ~100 kb unique haplotype relative to strains carried divergent haplotypes. The plot shows the boundaries of the longest shared haplotype (the range over which it is identical to at least one other haplotype) around the reference allele of the focal marker (chr01a_1495970) relative to the divergent allele.

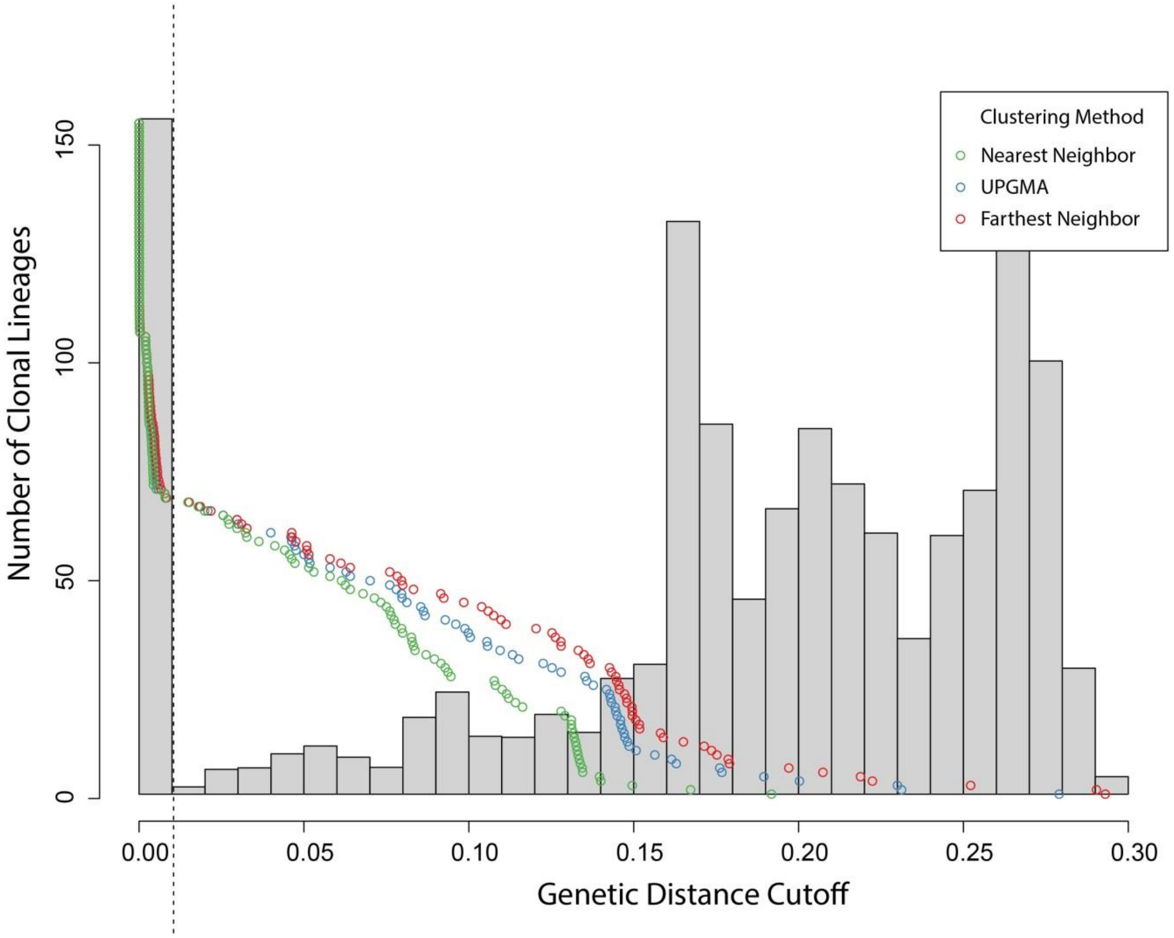

Supplementary Fig. 10. Graphical representation of three different clustering algorithms collapsing the 156 *Toxoplasma gondii* genomes into clonal lineages. The horizontal axis is the genetic distance based on a dissimilarity matrix as calculated in poppr R package. The vertical axis represents the number of lineages observed. Each point shows the threshold at which one would observe a given number of uncollapsed groups or individuals. The vertical dashed line marks the threshold used to collapse the 156 genomes into 10 clonal lineages and 59 non-clonal strains.

type I

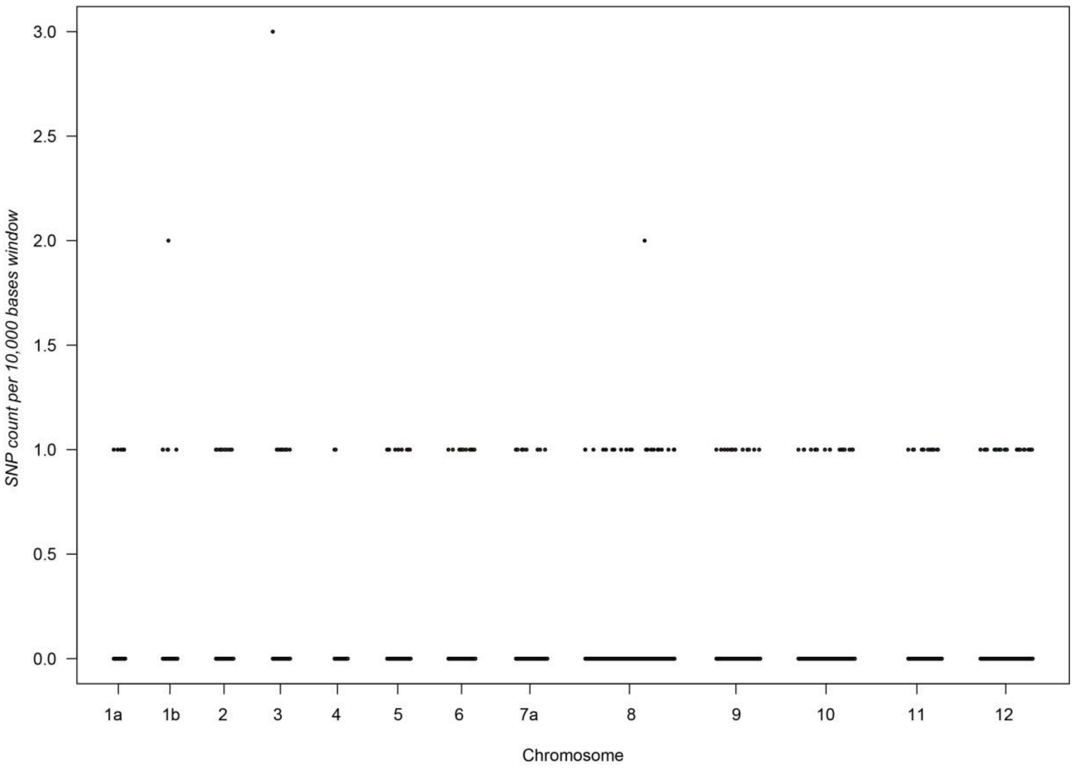

Supplementary Fig. 11a.

type II

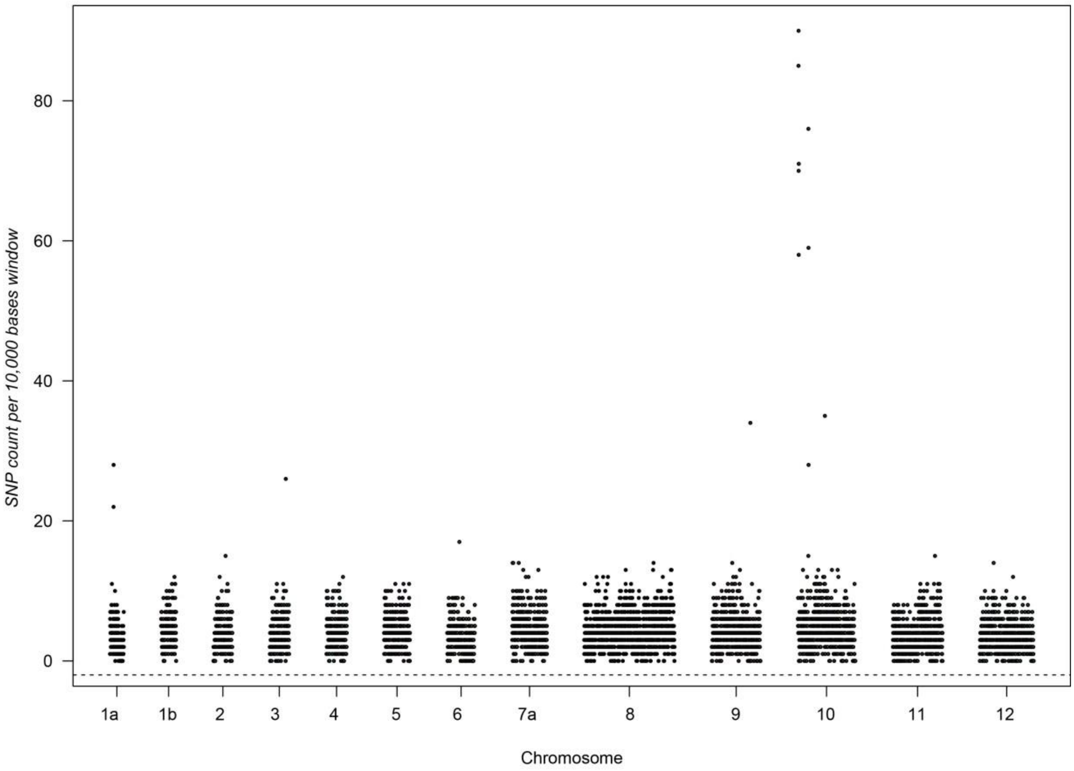

Supplementary Fig. 11b.

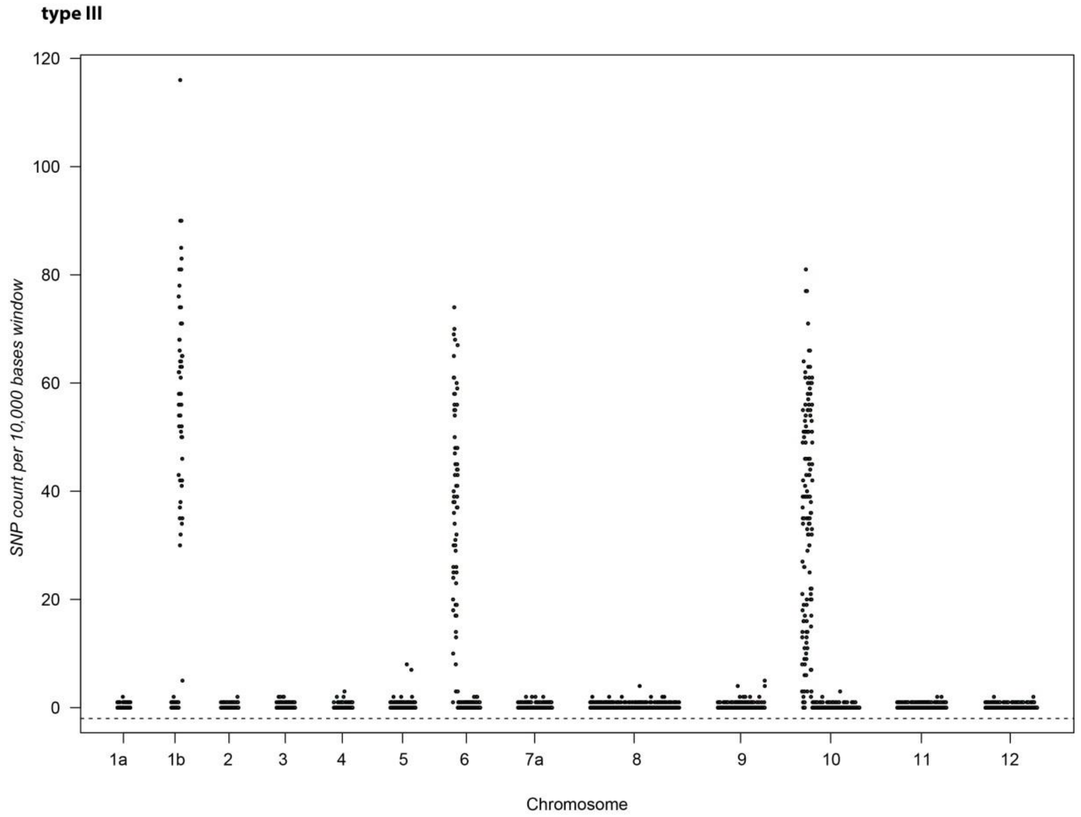

Supplementary Fig. 11c.

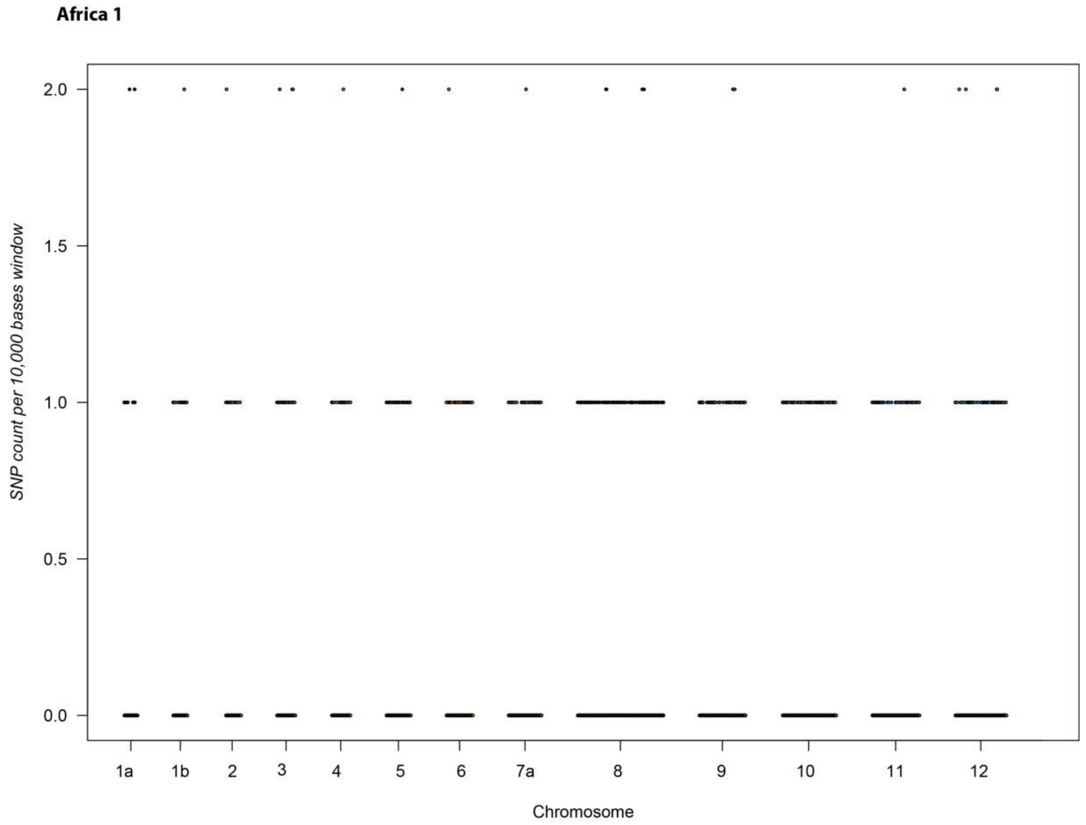

Supplementary Fig. 11d.

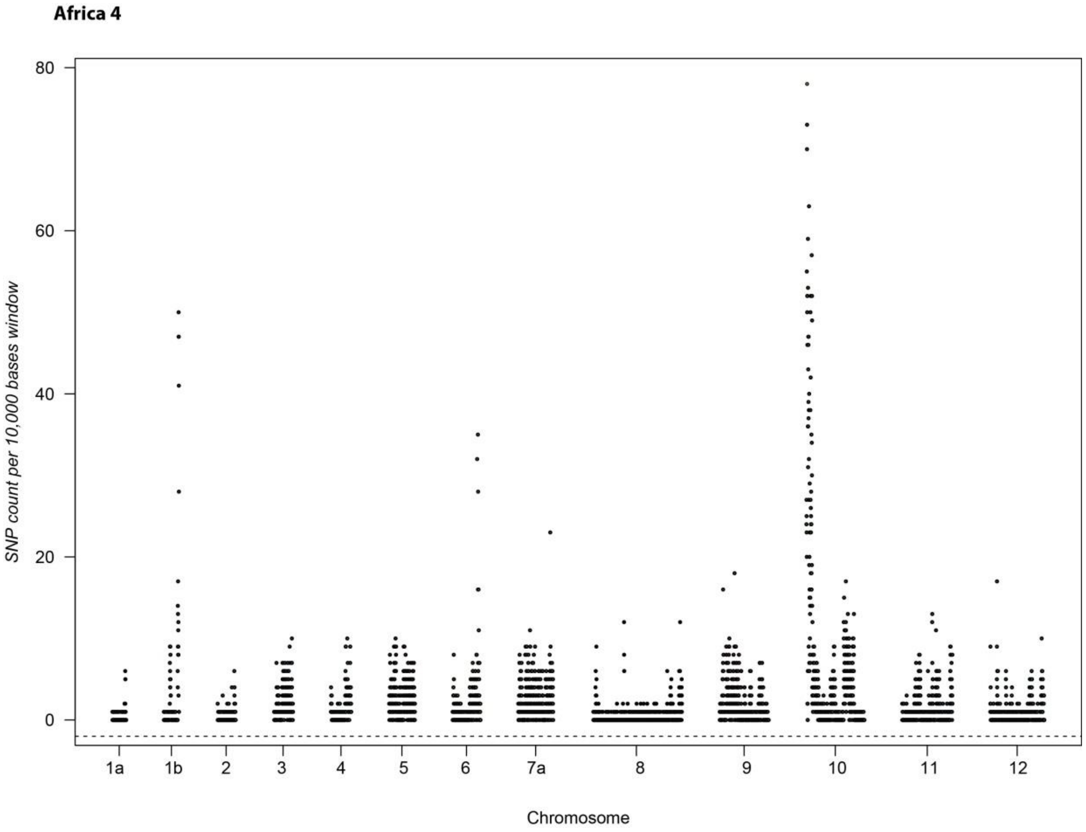

Supplementary Fig. 11e

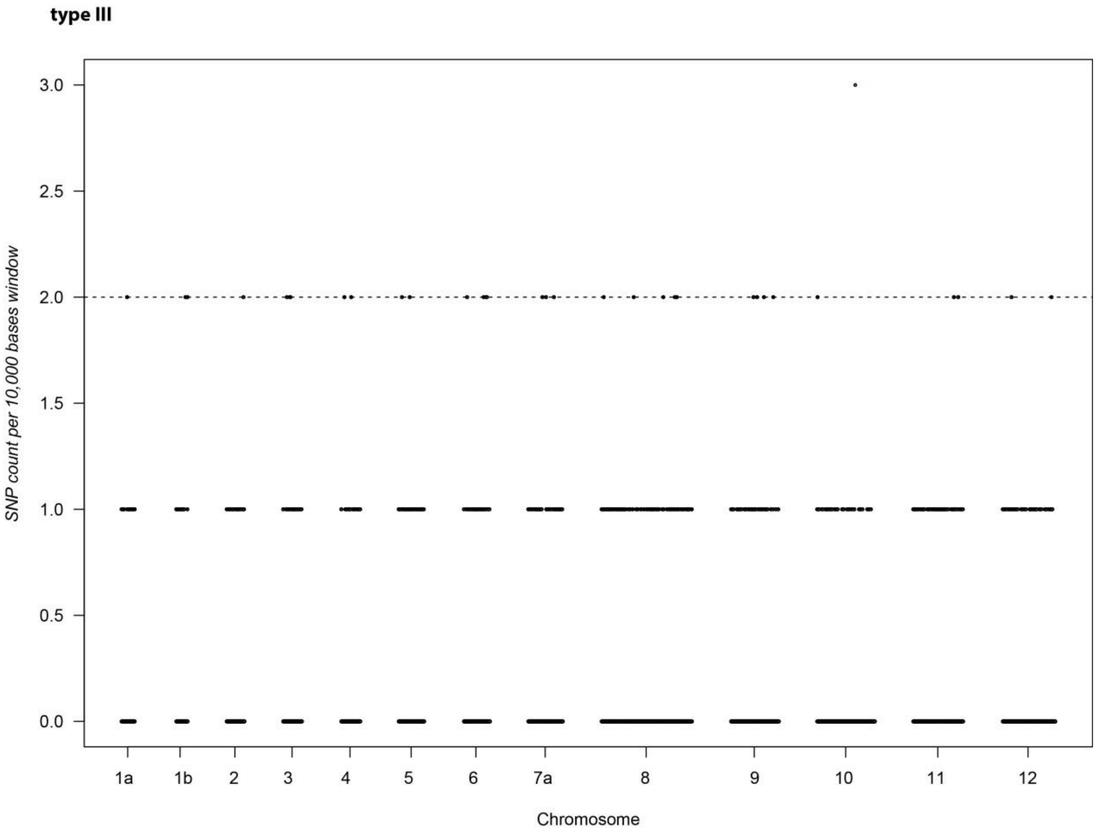

Supplementary Fig. 11f

Supplementary Fig. 11. SNPs density per window comparing strains of each intercontinental *poppr*-defined clonal lineages. Plots were generated for each of the five intercontinental lineages: type I (a), type II (b), type III (c), Africa 1 (d) and Africa 4 (e). A plot was generated for type III following the exclusion of USA04 genome (f). The total number of SNPs per 10 kb window for all strains within a lineage is plotted along the y-axis. Chromosome numbers are indicated along the x-axis.

Supplementary Table 1. **Amino acid substitutions on the top candidate gene for cat-adaptation TGRH88_020330 (TGME49_295920)**

ID	M	S	K	N	Y	D	Q	S	N	L
USA11				G	H	G		R	H	S
USA14				G	H	G		R	H	S
BENIN04				G	H	G		R	H	S
BRAZIL01				G	H	G		R	H	S
USA02				G	H	G		R	H	S
SPAIN01				G	H	G		R	H	S
PANAMA01				G	H	G		R	H	S
GABON08				G	H	G		R	H	S
GABON10				G	H	G		R	H	S
GABON04				G	H	G		R	H	S
GABON06				G	H	G		R	H	S
GUADELOUPE01				G	H	G		R	H	S
GUADELOUPE02				G	H	G		R	H	S
FRENCHGUIANA01				G	H	G		R	H	S
FRENCHGUIANA03				G	H	G		R	H	S
FRENCHGUIANA04				G	H	G		R	H	S
FRENCHGUIANA07				G	H	G		R	H	S
USA04				G	H	G		R	H	S
MARTINIQUE01				G	H	G		R	H	S
MARTINIQUE02				G	H	G		R	H	S
MARTINIQUE03				G	H	G		R	H	S
MARTINIQUE05				G	H	G		R	H	S
USA06				G	H	G		R	H	S
CHINA01				G	H	G		R	H	S
PORTUGAL09				G	H	G		R	H	S
PORTUGAL10				G	H	G		R	H	S
USA07				G	H	G		R	H	S
SENEGAL04			Q	G		G		R	H	S
SENEGAL11				G	H	G		R	H	S
SENEGAL23			Q	G		G		R	H	S
USA08				G	H	G		R	H	S
BRAZIL03				G	H	G		R	H	S
BRAZIL04				G	H	G		R	H	S
BRAZIL05				G	H	G		R	H	S
BRAZIL06				G	H	G		R	H	S
BRAZIL08				G	H	G		R	H	S
BRAZIL09				G	H	G		R	H	S
BRAZIL10				G	H	G		R	H	S
BRAZIL11				G	H	G		R	H	S
BRAZIL12				G	H	G		R	H	S
BRAZIL13				G	H	G		R	H	S
BRAZIL14				G	H	G		R	H	S
BRAZIL15				G	H	G		R	H	S
COSTARICA1				G	H	G		R	H	S
COSTARICA2				G	H	G		R	H	S
GUYANA01				G	H	G		R	H	S
COLOMBIA01				G	H	G		R	H	S
COLOMBIA02				G	H	G		R	H	S
COSTARICA3				G	H	G		R	H	S
USA09				G	H	G		R	H	S
USA12										
URUGUAY01										
CANADA01		Y								
FRENCHGUIANA15										
FRENCHGUIANA23										
FRENCHGUIANA24		Y					A			
FRENCHGUIANA09	I									
FRENCHGUIANA10										
FRENCHGUIANA18										
FRENCHGUIANA05										
FRENCHGUIANA19										
FRENCHGUIANA21	I									
FRENCHGUIANA11										
FRENCHGUIANA22										
FRENCHGUIANA20										
FRENCHGUIANA14										
FRENCHGUIANA08										
FRENCHGUIANA16		Y					A			
USA13										
FRENCHGUIANA17										
FRENCHGUIANA05										

Supplementary Table 2. Genetic diversity of the most common intercontinental clonal lineages

	Number of strains	π diversity*	SNP distance between the two most divergent strains within the clonal lineage with filtering out singletons	SNP distance between the two most divergent strains within the clonal lineage without filtering out singletons
Type I	3	5.6x10-5	160	502
Type II	48	1.1x10-4	8,408	9,116
Type III	19	1.7x10-5	350	457
Africa 1	11	2.6x10-5	453	489

*Average nucleotide diversity calculated from the division of the genome into 10kb-windows (VCFtools)

The Variant Call Format and VCFtools, Petr Danecek, Adam Auton, Goncalo Abecasis, Cornelis A. Albers, Eric Banks, Mark A. DePristo, Robert Handsaker, Gerton Lunter, Gabor Marth, Stephen T. Sherry, Gilean McVean, Richard Durbin and 1000 Genomes Project Analysis Group, Bioinformatics, 2011

Supplementary Discussion 1

We show that for certain chromosomes type II lineage has sister clades (close but distinct) among type 12 strains (USA11, USA12 and USA13). This was evident from global (Supplementary Fig. 1-2) and local ancestry analyses (Fig. 3), beside genetic divergence evaluation (Supplementary Fig. 6). Type 12 is constituted of at least two populations or lineages: (1) RFLP lineage #4, the most common lineage in the domestic environment of North America after type II and III and (2) RFLP lineage #5, the most common wild lineage on the same continent. RFLP lineage #5 (represented by USA12 and USA13) and type II, in spite of having chromosomes of the same ancestry, exhibit clear divergence from type II lineage (Supplementary Fig. 6). This pattern confirms that RFLP lineage #5 is a true wild lineage sharing no recent ancestry with type II lineage. RFLP lineage #4 (represented by USA11) showed an admixed pattern of ancestry consistent with a recent recombination between a type II and a strain of RFLP lineage #5. Evidence from apicoplast sequences also shows that Asian Chinese 1 shares a common ancestor with wild type 12. Note that all these strains belong to the same clade (refer to Supplementary Fig. 1). These data support the occurrence of *T. gondii* migrations between Asia and North America, probably anterior to the Neolithic revolution and the domestication era. Migrations were probably mediated by movements of animal herds through the land bridge formed by the Bering Strait during the late Pleistocene period until about 13,000 years ago. During this period a corridor was created by falling sea levels that provided an opportunity for Asian species including mammoths, bison, muskoxen, caribou, lions, brown bears, and wolves to move into North America [17–20]. Assuming a role of late Pleistocene animal species in disseminating *T. gondii*, an Asian origin of this clade appears more likely given the direction of migrations inferred for these animals. It is consistent with the hypothesis of an Old World origin of type II lineage, as previously suggested by Shwab et al. [21] using multilocus markers and not a North American origin as proposed by other studies [22,23]. USA14 (B73), although isolated in the wild (from a bear) had a chromosomal ancestry consistent with recent recombination having occurred between type II and type III lineages. This latter observation is consistent with evidence from multilocus markers of a dissemination of T. *gondii* strains from the domestic environment into the wild environment in North America[24].

Supplementary Discussion 2

In North America, the large majority of *T. gondii* strains isolated directly from the environment of domestic cats belong to either type II, type III RFLP lineages #4 or #8, and all carry the

unique ~100 kb haplotype of *T. gondii*[24]. Their prevalence diminishes with distance from the home range of domestic cats and they represent less than 40% of strains isolated in the wild. *T. gondii* strains carrying divergent haplotypes on the ~100 kb region of chromosome 1a exhibit the opposite pattern: RFLP lineage #5 represents 0.5% of North American domestic isolates and 48.8% of wild ones. On this continent, important populations of wild felids (mainly *Lynx rufus*) are still found[25,26]. In French Guiana, in South America, the environmental segregation between unique ~100 kb haplotype and divergent ones is very pronounced, which is probably explained by the relatively well preserved Amazonian environment in this region and the high density of wild felids in this environment[27–29]. The situation in other South American countries such as Brazil is closer to the situation in North America, since *T. gondii* strains carrying the unique ~100 kb haplotype have been often isolated from both domestic and wild hosts[30,31]. This observation is consistent with the substantial environmental degradation and decline in populations of wild felids observed in many areas on this continent[32].

Supplementary Discussion 3

The results provided by this study reveals that a unique *T. gondii* haplotype on chromosome 1a is tightly associated to domestic cats in time and space. This *T. gondii* haplotype therefore appears to carry an adaptation for efficient transmission by domestic cats. In this section, we discuss a number of alternative hypotheses to this model.

1) Host species other than domestic cats could be driving the selection of the unique *T. gondii* haplotype.

Various host species get infected by *T. gondii* and many of these hosts have today a global distribution (e. g. humans, livestock). However, few are able to transmit the parasite in an efficient way to truly exert a selective pressure on parasite populations. The few species of hosts in which parasite life cycle completion occurs are considered as "evolutionarily significant host (ESH)", and include the domestic cat and its main prey species (rodents, followed by birds)[33]. House mice, brown rats (*Rattus norvecigus*) and black rats (*Rattus rattus*) have undergone recent global expansion, but are not still found in certain areas where the unique ~100 kb haplotype of *T. gondii* is well-established[34–37]. Interspecific competition between these three ESH species has been repeatedly documented often resulting in distinct patterns of species occurrence in their invaded range[38–40]. In addition, the capacity of house mice and rats to transmit *T. gondii* depends on their ability to survive acute infection and develop persistent tissue-cysts the main source of infection for cats[41]. In mice, virulence of *T. gondii* shows no association with the ~100 genomic region on chromosome 1a, since many *T. gondii* strains carrying the unique ~100 kb haplotype cause lethal infections[42,43,28] while certain *T. gondii* strains carrying divergent haplotypes (e.g. RFLP lineage #5) cause chronic infections[21,44]. *T. gondii* virulence in rats is less documented although they appear highly resistant to acute toxoplasmosis[45,46]. For example, brown rats have been shown to survive low and moderate doses of oocysts from *T. gondii* strains isolated in both domestic and wild environments (with no knowledge about the haplotype they carry)[47]. Domestic cats are the only hosts among these ESHs, whose global geographic distribution correlates with the occurrence of the ~100 kb unique haplotype of *T. gondii* identified in our study. The world's population of domestic cats was estimated to be 600 million[48] making this *T. gondii* host one of the world's most numerous animals. Their ability to shed tens of millions of highly resistant oocysts that survive for months in the environment and spread

over long distances[49], beside their large population sizes, provides a tremendous advantage to this species in terms of transmission capacity and efficiency.

2) This unique *T. gondii* haplotype does not carry a specific adaptation to cats (*Felis* spp) but is rather probably adapted to all species of felidae family.

Evidence from nuclear and apicoplast genomes support the notion that New World hybrid populations found in the domestic environment populations of *T. gondii* are the result recombinations between the major intercontinental lineages (types I, II, III or Africa 1) and New World non-hybrid wild populations. One could expect that these hybrid strains would carry mixed genomic ancestries of these two groups. This is true for most genomic regions, except on the ~100 kb genomic region of chromosome 1a on which only one haplotype is found in all hybrid populations. In other terms, New World non-hybrid wild populations significantly contributed in the emergence of hybrid populations, except at the ~100 kb genomic region of chromosome 1a where only Old World ancestry is observed (Fig. 4 d). This pattern points to a role of this specific genomic region in the selection of *T. gondii* strains that persist in the domestic environment. It is therefore likely that hybrid populations that have inherited divergent haplotypes at this genomic region are counter-selected in the domestic environment. The inability of *T. gondii* strains carrying divergent haplotypes at this specific genomic region in establishing in the domestic environment despite their efficient transmission in the wild supports the hypothesis of a poor adaptation of these strains to transmission by *T. gondii* hosts present in this environment. In the New World, the domestic environment is a very recent ecological niche for *T. gondii* since no felidae species was virtually found within domestic settlements of South and North America before the recent introduction of the domestic cat from the Old World. We showed that the domestic cat is the most probable host involved in this selective process (see above).

3) Given its Old World origin, this unique *T. gondii* haplotype probably carry an adaptation to most Old World species of felidae family rather than a specific adaptation to cats (*Felis* spp).

Felidae family emerged in the Old World and felid species diversity is more important in the Old World in comparison to the New World. Therefore, one could expect an important diversity of *T. strains* to occur in the Old World. However, the reverse is noticed, especially at the ~100 kb genomic region of chromosome 1a for which *T. gondii* genetic diversity is markedly low among Old World strains. This low diversity in the Old World could also be explained by a founder effect, in consistency with the hypothesis of a New World origin of *T. gondii* as a species and a subsequent spread to the Old World in ancient times [50]. However, phylogenetic evidence from the specific analysis of ~100 kb genomic region supports that the Old World clade emerged independently from the New World one (Fig. 4 d) and the geographical origin of their common ancestor remains elusive at this stage. The hypothesis of a massive sweep of Old World *T. gondii* populations is therefore more likely, and could be explained by the collapse of most felidae species in Africa, Asia and Europe. Meanwhile, the domestic cat has greatly proliferated and could also have driven a major selective sweep of Old World *T. gondii* populations. During this process, the ~100 kb genomic region of chromosome 1a appears to have been under strong selective pressure given the very low diversity specifically

observed at this genomic region among Old World strains. This pattern could be the result of a process comparable to the process that took place during the recent emergence of hybrid populations in the New World following domestic cat introduction in the New World (see above). More sampling efforts could be necessary to identify other Old World haplotypes at the ~100 kb genomic region which could be associated to adaptation to persisting wild Old World felidae populations.

4) This unique *T. gondii* haplotype has an Old World origin, but emerged at least around 26,000 years ago and could have therefore spread to the New World via Bering Strait until about 13,000 years ago (see Supplementary Discussion 1) and not only in recent years.

Few opportunities were provided for *T. gondii* to undergo migrations between the Old and New Worlds since these migrations could only be driven by the migrations of *T. gondii* hosts. Large scale *T. gondii* hosts migrations took place via the Bering Strait until about 13,000 years ago and more recently since the onset of the European age of exploration. Migrations of this *T. gondii* haplotype between Old and New World are likely to have been mediated by the major lineages (types I, II, III or Africa 1) given their intercontinental occurrence. In a previous study, Shwab et al.[21] analysed a large global sample of type II strains using microsatellite markers. Their results suggested an Old World origin of type II lineage and a subsequent introduction in North America from Europe. Assuming a spread to the New World via Bering Strait, an introduction from Asia would have been more likely. The present study was the first to apply timing estimates to date migration events. Estimated divergence times between Old and New World strains of same ancestry at whole-genome and chromosome level were consistent with very recent migrations between these two geographic entities, supporting the hypothesis of migrations taking place since the onset of the European age of exploration rather than via the Bering Strait. Type I, type III and Africa 1 lineages are much more recent. TMRCA estimates support that they most probably emerged before the onset of the European age of exploration, but confidence intervals of these estimates do not fully exclude an emergence subsequent do the onset of this historical period. Therefore, we cannot rule out an emergence of these lineages in the New World from at least one recently introduced Old World ancestor carrying the unique *T. gondii* haplotype (most likely type II lineage).

Supplementary Discussion 4

From the beginning of the Iron age, about 3,000 years ago maritime transportation substantially increased in the Old World [51]. Maritime activities of Romans in Antiquity[52], of Phoenician during medieval times[53] and of Vikings between the 7th and the 11th centuries[54], contributed to the expansion of domestic cats, mice and rats throughout the Mediterranean basin and to Continental Europe. During these periods Egyptian domestic cats gradually took over other populations of domestic cats such as in Anatolia before spreading to most areas of the Old World[55]. These movements of *T. gondii* hosts could have promoted encounters with previously allopatric populations of *T. gondii* (e.g. between Europe and Africa) fostering the emergence of new lineages by hybridization. This hypothesis is supported by the shared ancestry that is observed on certain genomic regions between the most common lineages. Boyle et al.[56] showed that types I and III are respectively second- and first-generation offspring of a cross between a type II strain and one of two

unknown ancestral strains. The most probable scenario is that these lineages emerged following the expansion of the geographical range of type II strains in the Old World during these periods.

Supplementary Discussion 5

Most Old World and New World hybrid strains appear to be the results of only one or limited rounds of meiotic reproduction when considering the chromosomal pattern of ancestry of experimental hybrids[57]. Indeed, we did not observe a fine mosaic of different ancestries alternating across genomes, as is usually observed when sexual recombination often occurs in a population[58–60]. Sexual recombination in *T. gondii* is favoured by mixed infections in cat prey, which is limited by the immunity developed by an intermediate host following its primary infection (referred to in the Introduction). This immunity often protects the intermediate host from new infections with different strains, but not from highly divergent strains as found in South America[61,62]. We propose that following their introduction in the Americas, rodents infected with Old World lineages were exposed to highly divergent strains from the wild environment near human settlements. Their immunity being unable to contain these new infections, the rodents could have become superinfected with highly divergent strains giving rise to tissue-cysts of divergent strains alongside tissue-cysts emanating from their primary infection. This unique situation would provide favourable conditions for the emergence of hybrid populations due to cats feeding on these superinfected intermediate hosts. The emergence of big cities, the decline of wildlife and the great proliferation of domestic cats would have gradually limited the exposure of domestic intermediate hosts to wild strains.

REFERENCES

1. Milgroom, M. G. *Population biology of plant pathogens: genetics, ecology, and evolution*. (APS Press, The American Phytopathological Society, 2015).
2. Graham, J., McNeney, B. & Seillier-Moiseiwitsch, F. Stepwise detection of recombination breakpoints in sequence alignments. *Bioinformatics* **21**, 589–595 (2005).
3. Radke, J. R. *et al.* Defining the cell cycle for the tachyzoite stage of Toxoplasma gondii. *Molecular and biochemical parasitology* **115**, 165–175 (2001).
4. Djurković-Djaković, O. *et al.* Kinetics of parasite burdens in blood and tissues during murine toxoplasmosis. *Experimental parasitology* **131**, 372–376 (2012).
5. Jerome, M. E., Radke, J. R., Bohne, W., Roos, D. S. & White, M. W. Toxoplasma gondii bradyzoites form spontaneously during sporozoite-initiated development. *Infection and immunity* **66**, 4838–4844 (1998).

6. Dubey, J. P. Oocyst shedding by cats fed isolated bradyzoites and comparison of infectivity of bradyzoites of the VEG strain Toxoplasma gondii to cats and mice. *Journal of Parasitology* **87**, 215–219 (2001).
7. Dubey, J. P., Miller, N. L. & Frenkel, J. K. The Toxoplasma gondii oocyst from cat feces. *Journal of Experimental Medicine* **132**, 636–662 (1970).
8. Elmore, S. A. *et al.* Toxoplasma gondii: epidemiology, feline clinical aspects, and prevention. *Trends in parasitology* **26**, 190–196 (2010).
9. Lappin, M. R. Update on the diagnosis and management of Toxoplasma gondii infection in cats. *Topics in companion animal medicine* **25**, 136–141 (2010).
10. Freppel, W. *et al.* Structure, composition, and roles of the Toxoplasma gondii oocyst and sporocyst walls. *The Cell Surface* **5**, 100016 (2019).
11. Lélu, M. *et al.* Quantitative estimation of the viability of Toxoplasma gondii oocysts in soil. *Applied and environmental microbiology* **78**, 5127–5132 (2012).
12. Naughton, D. *The natural history of Canadian mammals*. (University of Toronto Press, 2012).
13. Phelan, J. P. & Austad, S. N. Natural selection, dietary restriction, and extended longevity. *Growth, development, and aging: GDA* **53**, 4–6 (1989).
14. König, B. & Markl, H. Maternal care in house mice. *Behavioral Ecology and Sociobiology* **20**, 1–9 (1987).
15. Solomon, N. G. Age of pairing affects reproduction in prairie voles. *Laboratory Animals* **25**, 232–235 (1991).
16. Kopelman, N. M., Mayzel, J., Jakobsson, M., Rosenberg, N. A. & Mayrose, I. Clumpak: a program for identifying clustering modes and packaging population structure inferences across K. *Molecular ecology resources* **15**, 1179–1191 (2015).
17. Guthrie, R. D. Radiocarbon evidence of mid-Holocene mammoths stranded on an Alaskan Bering Sea island. *Nature* **429**, 746–749 (2004).
18. Lowe, J. J. & Walker, M. *Reconstructing quaternary environments*. (Routledge, 2014).

19. Froese, D. *et al.* Fossil and genomic evidence constrains the timing of bison arrival in North America. *Proceedings of the National Academy of Sciences* **114**, 3457–3462 (2017).
20. Phillips, L., Chambers, N. & Backensto, S. Migration: On the move in Alaska. *Alaska Park Science* **17**, 1–3 (2018).
21. Shwab, E. K. *et al.* Human impact on the diversity and virulence of the ubiquitous zoonotic parasite Toxoplasma gondii. *Proceedings of the National Academy of Sciences* **115**, E6956–E6963 (2018).
22. Khan, A. *et al.* Recent transcontinental sweep of Toxoplasma gondii driven by a single monomorphic chromosome. *Proc. Natl. Acad. Sci. U.S.A.* **104**, 14872–14877 (2007).
23. Minot, S. *et al.* Admixture and recombination among Toxoplasma gondii lineages explain global genome diversity. *Proceedings of the National Academy of Sciences* **109**, 13458–13463 (2012).
24. Jiang, T. *et al.* A partition of Toxoplasma gondii genotypes across spatial gradients and among host species, and decreased parasite diversity towards areas of human settlement in North America. *International journal for parasitology* https://pubmed.ncbi.nlm.nih.gov/29577892/ (2018) doi:10.1016/j.ijpara.2018.01.008.
25. Kaczensky, P. *et al.* Status, management and distribution of large carnivores–bear, lynx, wolf & wolverine–in Europe. Document prepared with the assistance of Istituto di Ecologia Applicata and with the contributions of the IUCN/SSC Large Carnivore Initiative for Europe under contract N 070307. (2012).
26. Kelly, M., Morin, D. & Lopez-Gonzalez, C. A. *Lynx rufus. The IUCN Red List of Threatened Species. 2016: e. T12521A50655874*. (2019).
27. Hammond, D. S. *Tropical forests of the Guiana Shield: ancient forests in a modern world*. (CABI, 2005).
28. Mercier, A. *et al.* Human impact on genetic diversity of Toxoplasma gondii: example of the anthropized environment from French Guiana. *Infect. Genet. Evol.* **11**, 1378–1387 (2011).

29. De Thoisy, B. *et al.* Predators, prey and habitat structure: can key conservation areas and early signs of population collapse be detected in neotropical forests? *PLoS One* **11**, e0165362 (2016).

30. Pena, H. F. J. *et al.* Isolation and genetic characterisation of Toxoplasma gondii from a red-handed howler monkey (Alouatta belzebul), a jaguarundi (Puma yagouaroundi), and a black-eared opossum (Didelphis aurita) from Brazil. *Veterinary parasitology* **175**, 377–381 (2011).

31. Cabral, A. D. *et al.* First isolation and genotyping of Toxoplasma gondii from bats (Mammalia: Chiroptera). *Veterinary parasitology* **193**, 100–104 (2013).

32. Bullock, E. L., Woodcock, C. E., Souza Jr, C. & Olofsson, P. Satellite-based estimates reveal widespread forest degradation in the Amazon. *Global Change Biology* **26**, 2956–2969 (2020).

33. Müller, U. B. & Howard, J. C. The impact of Toxoplasma gondii on the mammalian genome. *Current opinion in microbiology* **32**, 19–25 (2016).

34. Dalecky, A. *et al.* Range expansion of the invasive house mouse M us musculus domesticus in S enegal, W est A frica: a synthesis of trapping data over three decades, 1983–2014. *Mammal Review* **45**, 176–190 (2015).

35. Galal, L. *et al.* The introduction of new hosts with human trade shapes the extant distribution of Toxoplasma gondii lineages. *PLoS Negl Trop Dis* **13**, e0007435 (2019).

36. Hima, K. *et al.* Native and invasive small mammals in urban habitats along the commercial axis connecting Benin and Niger, West Africa. *Diversity* **11**, 238 (2019).

37. Hamidović, A. *et al.* A hotspot of Toxoplasma gondii Africa 1 lineage in Benin: How new genotypes from West Africa contribute to understand the parasite genetic diversity worldwide. *PLoS neglected tropical diseases* **15**, e0008980 (2021).

38. Yom-Tov, Y., Yom-Tov, S. & Moller, H. Competition, coexistence, and adaptation amongst rodent invaders to Pacific and New Zealand islands. *Journal of Biogeography* **26**, 947–958 (1999).

39. Lack, J. B., Hamilton, M. J., Braun, J. K., Mares, M. A. & Van Den Bussche, R. A. Comparative phylogeography of invasive Rattus rattus and Rattus norvegicus in the US reveals distinct colonization histories and dispersal. *Biological invasions* **15**, 1067–1087 (2013).

40. Feng, A. Y. & Himsworth, C. G. The secret life of the city rat: a review of the ecology of urban Norway and black rats (Rattus norvegicus and Rattus rattus). *Urban Ecosystems* **17**, 149–162 (2014).

41. Dubey, J. P. Comparative infectivity of oocysts and bradyzoites of Toxoplasma gondii for intermediate (mice) and definitive (cats) hosts. *Veterinary parasitology* **140**, 69–75 (2006).

42. Pena, H. F. J., Gennari, S. M., Dubey, J. P. & Su, C. Population structure and mouse-virulence of Toxoplasma gondii in Brazil. *Int. J. Parasitol.* **38**, 561–569 (2008).

43. Mercier, A. *et al.* Additional haplogroups of Toxoplasma gondii out of Africa: population structure and mouse-virulence of strains from Gabon. *PLoS Negl Trop Dis* **4**, e876 (2010).

44. Howe, D. K. & Sibley, L. D. Toxoplasma gondii comprises three clonal lineages: correlation of parasite genotype with human disease. *J. Infect. Dis.* **172**, 1561–1566 (1995).

45. FUJII, H., KAMIYAMA, T. & HAGIWARA, T. Species and strain differences in sensitivity to Toxoplasma infection among laboratory rodents. *Japanese Journal of Medical Science and Biology* **36**, 343–346 (1983).

46. Ruffolo, B. B. *et al.* Isolation and genotyping of toxoplasma gondii in seronegative urban rats and presence of antibodies in communicating dogs in Brazil. *Revista do Instituto de Medicina Tropical de Sao Paulo* **58**, (2016).

47. Dubey, J. P. *et al.* Experimental toxoplasmosis in rats induced orally with eleven strains of Toxoplasma gondii of seven genotypes: tissue tropism, tissue cyst size, neural lesions, tissue cyst rupture without reactivation, and ocular lesions. *PloS one* **11**, e0156255 (2016).

48. Baker, P. J., Soulsbury, C. D., Iossa, G. & Harris, S. Domestic cat (Felis catus) and domestic dog (Canis familiaris). (2010).

49. Frenkel, J. K., Dubey, J. P. & Miller, N. L. Toxoplasma gondii in cats: fecal stages identified as coccidian oocysts. *Science* **167**, 893–896 (1970).

50. Bertranpetit, E. *et al.* Phylogeography of Toxoplasma gondii points to a South American origin. *Infect. Genet. Evol.* **48**, 150–155 (2017).

51. Jones, E. P., Eager, H. M., Gabriel, S. I., Jóhannesdóttir, F. & Searle, J. B. Genetic tracking of mice and other bioproxies to infer human history. *Trends in Genetics* **29**, 298–308 (2013).
52. Peters, J. *Römische Tierhaltung und Tierzucht: eine Synthese aus archäozoologischer Untersuchung und schriftlich-bildlicher Überlieferung*. vol. 5 (Leidorf, 1998).
53. Bonhomme, F. *et al.* Genetic differentiation of the house mouse around the Mediterranean basin: matrilineal footprints of early and late colonization. *Proceedings of the Royal Society B: Biological Sciences* **278**, 1034–1043 (2011).
54. Jones, E. P. *et al.* Fellow travellers: a concordance of colonization patterns between mice and men in the North Atlantic region. *BMC Evolutionary Biology* **12**, 1–8 (2012).
55. Ottoni, C. *et al.* The palaeogenetics of cat dispersal in the ancient world. *Nature Ecology & Evolution* **1**, 1–7 (2017).
56. Boyle, J. P. *et al.* Just one cross appears capable of dramatically altering the population biology of a eukaryotic pathogen like Toxoplasma gondii. *Proceedings of the National Academy of Sciences* **103**, 10514–10519 (2006).
57. Khan, A. *et al.* NextGen sequencing reveals short double crossovers contribute disproportionately to genetic diversity in Toxoplasma gondii. *BMC genomics* **15**, 1–15 (2014).
58. Henn, B. M. *et al.* Genomic ancestry of North Africans supports back-to-Africa migrations. *PLoS Genet* **8**, e1002397 (2012).
59. Fitak, R. R., Rinkevich, S. E. & Culver, M. Genome-wide analysis of SNPs is consistent with no domestic dog ancestry in the endangered Mexican wolf (Canis lupus baileyi). *Journal of Heredity* **109**, 372–383 (2018).
60. Kim, K. *et al.* The mosaic genome of indigenous African cattle as a unique genetic resource for African pastoralism. *Nature Genetics* **52**, 1099–1110 (2020).
61. Elbez-Rubinstein, A. *et al.* Congenital toxoplasmosis and reinfection during pregnancy: case report, strain characterization, experimental model of reinfection, and review. *J. Infect. Dis.* **199**, 280–285 (2009).

62. Jensen, K. D. *et al.* Toxoplasma gondii superinfection and virulence during secondary infection correlate with the exact ROP5/ROP18 allelic combination. *MBio* **6**, e02280-14 (2015).
