# Supplementary material for: A unique Toxoplasma gondii haplotype accompanied the global expansion of cats
Source: Nat Commun. 2022 Oct 1;13:5778. doi: 10.1038/s41467-022-33556-7 (PMC9526699; doi:10.1038/s41467-022-33556-7)
Supplement: Supplementary file 2 — Description of Additional Supplementary Files [file 41467_2022_33556_MOESM2_ESM.pdf]

Title: Supplementary Data 1. *Toxoplasma gondii* strains description

Description: hosts, geographical origins, microsatellite genotypes and whole-genome sequencing depth.

Title: Supplementary Data 2. Candidate missense variants associated to adaptation to cats.

Description: variants of putative functional relevance (missense) segregating intercontinental lineages and hybrid strains derived from intercontinental lineages from others populations.

Title: Supplementary Data 3. Candidate genes associated to adaptation to cats.

Description: gene selection is based on (1) presence of missense (nonsynonymous) variants specific to intercontinental lineages and hybrids; (2) expression, increased expression, or specific expression during enteroepithelial stages (EES) of development (early cellular forms characteristic of the onset of the sexual stage in cat enterocytes); and (3) degree of conservation in comparison to orthologs from the closest species to *T. gondii* (*Hammondia* or *Neospora*).

Title: Supplementary Data 4. Microsatellite markers and PCR primers used for the multiplex PCR assay
